# The Development of Recommendations for Healthcare Providers to Support Patients Experiencing Medication Self-Management Problems

**DOI:** 10.3390/healthcare11111545

**Published:** 2023-05-25

**Authors:** Laura Mortelmans, Eva Goossens, Anne-Marie De Cock, Mirko Petrovic, Patricia van den Bemt, Tinne Dilles

**Affiliations:** 1 Centre for Research and Innovation in Care (CRIC), Nurse and Pharmaceutical Care (NuPhaC), Department of Nursing Science and Midwifery, Faculty of Medicine and Health Sciences, University of Antwerp, 2610 Antwerp, Belgium; 2 Research Foundation Flanders (FWO), 1000 Brussels, Belgium; 3 Department of Public Health and Primary Care, KU Leuven, 3000 Leuven, Belgium; 4 Department of Patient Care, Antwerp University Hospital (UZA), 2610 Antwerp, Belgium; 5 Department of Geriatrics, ZNA, 2020 Antwerp, Belgium; 6 Department of Family Medicine and Population Health, University of Antwerp, 2610 Antwerp, Belgium; 7 Department of Geriatrics, Ghent University Hospital, 9000 Ghent, Belgium; 8 Section of Geriatrics, Department of Internal Medicine and Paediatrics, Ghent University, 9000 Ghent, Belgium; 9 Department of Clinical Pharmacy and Pharmacology, University Medical Center Groningen, 9713 GZ Groningen, The Netherlands

**Keywords:** medication therapy management, self-management, polypharmacy, Delphi technique

## Abstract

Medication self-management problems such as the inability to correctly obtain, understand, organize, administer or monitor medication can result in negative patient outcomes. However, supportive tools for healthcare providers to assist patients with medication self-management problems are lacking. This study aimed to develop recommendations for healthcare providers to support patients with polypharmacy who experience medication self-management problems. A three-phase study was conducted starting with (1) the mapping of medication self-management problems, followed by (2) a scoping review providing a list of relevant interventions and actions for each respective problem and (3) a three-round modified e-Delphi study with experts to reach consensus on the relevance and clarity of the recommended interventions and actions. The cut-off for consensus on the relevance and clarity of the recommendations was set at 80% expert agreement. Experts could propose additional recommendations based on their professional experience and expertise. The experts (n = 23) involved were healthcare professionals (i.e., nurses, pharmacists, and physicians) with specific expertise in medication management of patients with polypharmacy. Simultaneous with the second e-Delphi round, a panel of patients with polypharmacy (n = 8) evaluated the usefulness of recommendations. Results obtained from the patient panel were fed back to the panel of healthcare providers in the third e-Delphi round. Descriptive statistics were used for data analysis. Twenty medication self-management problems were identified. Based on the scoping review, a list of 66 recommendations for healthcare providers to support patients with the identified medication self-management problems was composed. At the end of the three-round e-Delphi study, the expert panel reached consensus on the relevance and clarity of 67 recommendations, clustered according to the six phases of the medication self-management model by Bailey et al. In conclusion, this study resulted in a guidance document including recommendations that can serve as a resource for healthcare providers to support patients with polypharmacy in case of medication self-management problems. Future research should focus on the evaluation of the feasibility and user-friendliness of the guide with recommendations in clinical practice.

## 1. Introduction

Patient-centered care, empowerment and self-management are increasingly encouraged in current healthcare systems [1,2]. Self-management, as part of self-care, is gaining importance, especially given the growing number of people with multiple chronic conditions [2,3].

Self-management of medication involves patients performing a range of actions, requiring knowledge, skills and behaviors, to manage their medication regimen as prescribed [4]. According to the medication self-management model of Bailey et al. (2013), this process starts with picking up prescribed medication at the pharmacy and filling prescriptions. Furthermore, medication self-management requires patients to have knowledge of their medication and to be able to understand the medication schedule, organize and plan daily medication intake, take the prescribed medicines, monitor their medication intake, and evaluate possible side effects or problems regarding their medication. The final step consists of maintaining a safe and effective daily medication intake routine as long as indicated [4]. 

Patients often experience problems regarding medication self-management, which affects medication adherence and safe medication use. A systematic review showed that 17% of patients with chronic diseases did not pick up their medicines from the pharmacy [5]. Furthermore, a study on patients with polypharmacy showed that 48% did not have the required medicines available, 31% did not have the necessary prescriptions and about 3% did not have the resources available to fill their prescriptions (e.g., no help from others, no transport) [6]. Patients experience deficiencies in understanding medication-related instructions or information [7,8,9] and in medication knowledge including not knowing the medication name, dosing or indication [6,10,11,12,13]. In addition, problems have been reported in organizing correct and timely intake of medication [14], opening medication packages [15,16,17,18,19,20] or storage of medicines [14,21,22]. The medication intake itself also proves problematic: taking an incorrect dose, taking the wrong medicine, forgetfulness or deliberately not taking medicines are common problems reported [6,23]. Patients’ knowledge of side effects and interactions seems insufficient [8,12]. If patients experience adverse effects, they do not always take appropriate actions, but rather abruptly stop their medication intake or self-adjust their medication regimen [6]. Moreover, patients sometimes reduce the use of their medicines over time or discontinue them prematurely [14,24,25].

Identifying and addressing problems in medication self-management during hospitalization is an opportunity to prevent medication-related problems after discharge and promote medication adherence and safe medication use at home [26]. Healthcare providers and patients have highlighted the importance of measures to support patients in medication self-management [27]. Training and supporting patients in the performance of medication self-management, as a partner in care, is recommended so patients feel ready to be responsible for the management of their medication after discharge, thereby maximizing therapeutic effects and minimizing side effects. Patients with polypharmacy may particularly benefit from medication self-management support, given the larger number of medicines and the more complex medication regimen [28]. 

However, in-hospital preparation and support for medication self-management proved to be inadequate [6]. Supportive tools for healthcare providers guiding patients through problems with medication self-management are lacking. Therefore, this study aimed to develop recommendations for healthcare providers to support patients with polypharmacy who experience medication self-management problems.

## 2. Materials and Methods

### 2.1. Study Design

**Figure 1:**
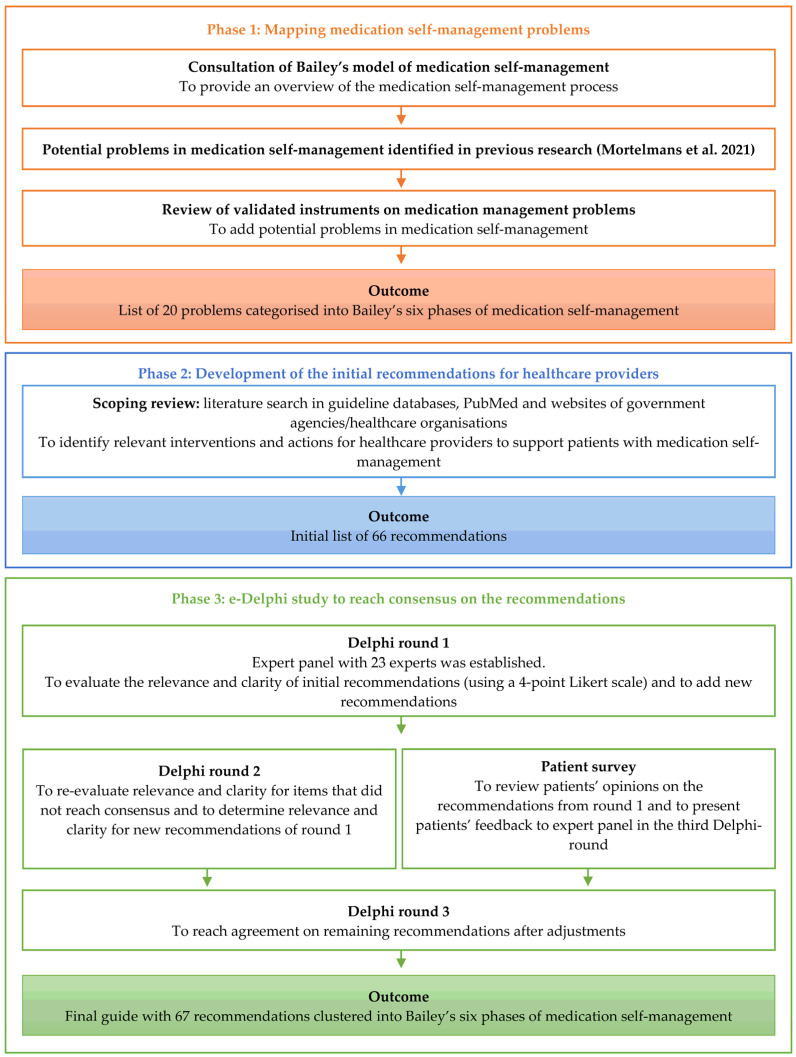
Study design of the development of recommendations for healthcare providers supporting patients with medication self-management problems.

A three-phase study was performed to develop recommendations for healthcare providers supporting polypharmacy patients with problems in medication self-management. Several components were performed, including (1) mapping of medication self-management problems, (2) a scoping review providing an overview of relevant interventions and actions for each respective problem and (3) a three-round e-Delphi study with experts to reach consensus on the relevance and clarity of the recommended interventions and actions. The steps are illustrated in Figure 1 and are explained below.

### 2.2. Phase One: Mapping of Medication Self-Management Problems

In the first phase, a list of frequently encountered medication self-management problems was compiled. The medication self-management model developed by Bailey et al. (2013) served as a framework, dividing the medication self-management process and related problems into six phases [29]. A recently conducted cross-sectional study investigating medication self-management deficiencies after hospital discharge was used to identify common problems encountered by patients with polypharmacy [6]. Furthermore, to complete the mapping of medication self-management problems, validated tools for assessing medication management problems were consulted [30,31,32,33].

### 2.3. Phase Two: Scoping Review

**Figure 2:**
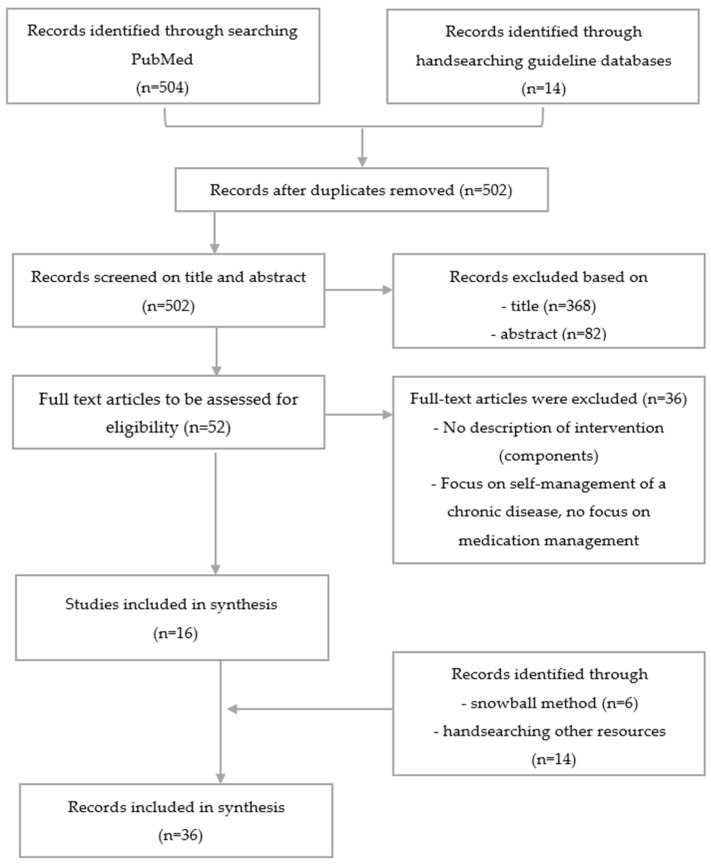
Selection of sources of evidence.

A scoping review was performed to provide an overview of interventions and actions to support patients with medication self-management problems. The selection process and results are reported in a flow diagram according to the PRISMA reporting guidelines (Figure 2) [34]. 

First, the National Institute for Health and Care Excellence (NICE), Guideline International Network (GIN), Scottish Intercollegiate Guidelines Network (SIGN), guidelines of the Dutch College of General Practitioners (NHG), the Dutch Guideline Database, Clinical Practice Guidelines (CPG), EBPracticeNet and the Belgian Health Care Knowledge Centre were manually reviewed for recommendations to support medication management. 

Consequently, PubMed was searched through combinations of Medical Subject Headings (MeSH terms) and free text terms related to the different phases of the medication self-management process and the self-management problems listed in the first phase (Appendix A Table A1). Guidelines and systematic reviews published between 2012 and 2022 in English, French or Dutch were eligible. The final search results were exported to Endnote X9, and duplicates were removed. The search results were screened by title and abstract for relevance. To be included, the abstract had to clearly report on interventions or actions intended to improve (elements of) medication management of adult patients. Subsequently, the full text of articles was reviewed, and successful interventions or actions described were extracted. 

Finally, records from guideline databases and PubMed were supplemented with other resources. For this purpose, websites of government agencies and healthcare organizations were manually reviewed (e.g., Federal Agency for Medicines and Health Products, National Institute for Health and Disability Insurance, the Dutch Institute for Rational Use of Medicine). 

Based on the interventions and actions found in the literature, a list of recommendations was composed. The recommendations were categorized according to the six phases of Bailey’s medication self-management model [4]. The list of recommendations was supplemented with background information on the medication self-management model and problems with medication self-management encountered by patients, the purpose of the guide, end users, target patient population and methodology. To validate this guide with recommendations, a modified e-Delphi study was conducted. 

### 2.4. Phase Three: A Modified e-Delphi Study

A Delphi study is a structured consensus method that uses a group facilitation technique, consisting of multiple survey rounds. This technique is used when the aim is to transform individual experts’ opinions into a single group consensus [35,36,37,38]. Instead of a classical Delphi method, characterized by a first round of open-ended questions, a modified Delphi was used. Panelists were provided with a set of pre-selected items, drawn from the scoping review, and were asked to quantitatively assess the relevance and clarity of these predefined items [39,40]. Since the administration of surveys was performed by email, the study is called an e-Delphi [40].

#### 2.4.1. Sample 

Experts were selected through purposive sampling. Eligibility criteria were as follows: (1) being a nurse, physician or pharmacist; (2) being employed in Belgium or The Netherlands; (3) having an established (inter)national profile in the field of medication management/patients with polypharmacy, recognized by scientific publications, policy reports and/or extensive participation in specialized thematic conferences, meetings or interest groups, or being involved in medication management of patients with polypharmacy in daily practice, as potential users of the guide; (4) having good Dutch language skills to complete the survey rounds. 

Experts were recruited at university/general hospitals, community healthcare, university colleges and faculties of medicine and health sciences or pharmaceutical sciences of universities. A balanced representation of all professional groups and settings (academic versus clinical practice) was pursued during the selection process. The number of experts needed to participate in the e-Delphi study did not depend on statistical power, as representativeness in such studies is assessed according to the quality of the sample rather than its size [41]. 

#### 2.4.2. e-Delphi Procedure

Experts received an email explaining the objectives of the study and were asked for consent to participate prior to the first e-Delphi round. Non-responders received up to two reminders. After expert consent was obtained, the distribution of Excel surveys and collection of data were performed by email. Written instructions for filling out the Excel file were provided. Participants were given four weeks to complete each survey form. Two reminder emails were sent to non-responders during each round. Each round was followed by a three- to four-week period for analyzing responses and integrating results into the next e-Delphi round. After each round, experts were provided with a synthesis document indicating the median score; agreement percentage; and decision on acceptance, removal or modification of an item. An instructional video clarified the content and interpretation of this document. The e-Delphi study was conducted between June and November 2022. 

##### Round 1

During the first e-Delphi-round (1 June–6 July 2022), experts were asked to perform the following actions:

1. Provide information on their demographics, professional background and specific area(s) of expertise for descriptive purposes and to confirm their eligibility in the first survey round.
2. Rate the level of relevance and clarity of each recommendation, using a 4-point Likert scale (1 = not relevant/not clear at all; 4 = very relevant/very clear). For each statement, participants were given the option to select ‘is not within my area of expertise’ as an alternative response. Free text space was provided, giving participants the opportunity to propose changes to the original recommendations, propose new recommendations based on their professional experience and expertise or emphasize their rating.
3. Rate the relevance of including the background information within the guide as well as the clarity of this information.
4. Assess different formats of the guide with recommendations (e.g., digital or paper version, use of index cards, tabular form) for usability and feasibility in clinical practice, using a 4-point Likert scale. This was intended to identify the most appropriate format of the guidance document to implement the recommendations in practice.

Descriptive statistics were used to describe participants’ characteristics and group responses to each statement using Microsoft Excel. Consensus for both relevance and clarity was defined as ≥80% of the experts assigned a score of 3 or 4 to a recommendation, which led to item retainment. If ≥80% of respondents assigned a score of ≤2, the recommendation was removed from the item list. For each item not reaching consensus, experts’ comments were used to revise the respective recommendations. Reformulated or modified recommendations were presented to the experts in the next survey round. 

##### Round 2

The second survey round (10 August–16 September 2022) consisted of a modified item list, including the initial recommendations for which no consensus was reached and new recommendations from the first round. Experts were invited to (1) re-evaluate relevance and clarity and add comments for items that did not reach consensus on relevance in the first round and (2) assign a score for the relevance and clarity of each new item. 

Simultaneously with the second e-Delphi round, recommendations were presented to a panel of patients with polypharmacy. Patients were purposefully recruited from a cardiology ward, the Multidisciplinary Oncological Centre, and the cystic fibrosis (CF) center of a Belgian University hospital. Patients admitted to these wards often take multiple chronic medicines for their condition. Patients with CF were specifically chosen given the higher likelihood of inclusion of young adults. Eligibility criteria were as follows: (1) being ≥18 years, (2) polypharmacy (i.e., taking ≥5 chronic medicines daily), (3) self-managing medication at home and (4) being Dutch-speaking. The eligibility of patients was assessed in consultation with the nurse pathway supervisors of the respective departments. In selecting participants, the aim was to have a heterogeneous panel in terms of age, gender, education and type of chronic conditions.

Socio-demographic data were surveyed to describe sample characteristics and confirm the eligibility of patients to participate in this study. Patients’ overall impression of recommendations was surveyed using statements. Patients were asked to mark each recommendation as useful or not useful, using green and red markers, respectively. If patients had no opinion on a particular recommendation, they had to leave the recommendation blank. Open-ended questions were used to identify which recommendations patients might not have considered useful, along with their reasoning. Subsequently, patients had the opportunity to suggest new recommendations. Space was provided for additional comments on the guidance document. As patients need to comprehend the content of the recommendations to ensure a proper evaluation, the extent to which patients found it difficult to understand the recommendations was probed. Surveys were administered face-to-face, so that, if necessary, additional information could be gathered. The duration of the face-to-face surveys ranged from 45 min to 1 h30. Patients’ feedback and suggestions were summarized in a narrative report and fed back to the expert panel of healthcare providers during the third e-Delphi round.

##### Round 3

In the third round (11 October–7 November 2022), consolidated feedback was presented for all items on which consensus on relevance or clarity was not reached in previous rounds. Furthermore, items that reached consensus on relevance among experts but were considered not meaningful by ≥80% of patients in the second round (or vice versa) were re-presented to the expert panel of healthcare providers during the third round. Experts had the opportunity to re-evaluate the items accordingly with (1) new relevance and clarity scores and (2) the formulation of comments (optional).

The number of survey rounds was set at three to prevent research fatigue. Three survey rounds also allowed free-text responses from Round 1 to be incorporated as new statements in Round 2 and re-evaluated based on the group consensus in Round 3. Recommendations with a lack of consensus after three rounds were removed.

### 2.5. Translation

For publication purposes, the Dutch version of the guide with recommendations was translated into English by a native English speaker of the University of Antwerp’s language institute (Linguapolis). The translator was not involved in this study.

## 3. Results

### 3.1. Phase One: Mapping of Medication Self-Management Problems

Twenty potential medication self-management problems were identified and categorized into Bailey’s six phases of medication self-management (Table 1).

### 3.2. Phase Two: Scoping Review

After removing duplicates, 502 articles were identified from guideline databases and PubMed. Based on the title and abstract, 450 articles were excluded. A total of 52 articles were included for full-text review. Sixteen articles were considered eligible. These were supplemented by 21 records identified through handsearching other resources, resulting in 36 records being included in the synthesis (Figure 2). A summary of the included records can be found in Supplementary File S3. Based on the interventions and actions found literature, a list of 66 recommendations was composed.

### 3.3. Phase Three: e-Delphi Study

Of 41 experts invited to participate in the e-Delphi study, a total of 23 experts confirmed participation and completed the first round (56.1% response rate). In the second round, 21 of the 23 experts who participated in the first round completed the survey (91.3% response rate), and 20 experts completed the third round (86.9% response rate). Participants’ characteristics are presented in Table 2. 

#### 3.3.1. Round 1

Consensus on both relevance and clarity was achieved for 44% (29) of 66 statements. In this first round, consensus was reached for more than 50% of recommendations in the domains of problems with organizing and planning medication use; medication intake; and monitoring side effects, risks and warnings (Appendix A Table A2). A total of 14 new recommendations were added, and 26 recommendations were rephrased based on experts’ feedback. Furthermore, 13 recommendations were combined, and 2 recommendations were removed due to overlap (Appendix A Table A3). 

Consensus was reached on the relevance of all aspects of the background information presented alongside the recommendations (i.e., the medication self-management model and problems with medication self-management encountered by patients, the purpose of the guide, end users, target patient population and methodology). All aspects were, therefore, retained. In terms of clarity, the objective of the guidance document and intended patient population failed to reach consensus (79%) and were reformulated based on experts’ comments. The initial focus on patient support was extended to patient and (informal) caregiver support.

After evaluating various guide formats and recommendations, a consensus was reached that an online/electronic tool would be the most user-friendly and feasible option for practical use.

#### 3.3.2. Round 2

Consensus was achieved for 79% (56) of 71 recommendations. In Round 2, 100% consensus was reached for recommendations in the domain of sustaining safe and appropriate medication use through the duration of the prescription (Appendix A Table A2). Fourteen recommendations were rephrased, two were differentiated between Belgian and Dutch context, three recommendations were split into more than one recommendation, and one was removed due to consensus on irrelevance (Appendix A Table A3).

Simultaneously with the second e-Delphi round among experts, eight patients provided feedback on the guide with recommendations. Patients’ characteristics are presented in Table 3. Seven patients indicated that recommendations were (very) easy to understand, while one patient rated the difficulty in understanding the recommendations as neutral. All patients found the guide useful. All participants agreed with the statement that the recommendations are meaningful to healthcare providers helping patients who experience problems with their medicines. Moreover, patients felt that healthcare providers can help with advice from the guide if they experience problems with their medicines. Patients indicated that the guide is important for healthcare providers to properly support patients in their medication self-management problems. Only 5 of 71 recommendations were marked as not helpful by one person each time. 

#### 3.3.3. Round 3

In the third e-Delphi round, consensus on 92% (68) of 74 recommendations was reached (Appendix A Table A2): sixty-seven recommendations on both relevance and clarity to be retained, one on irrelevancy to be removed from the list. The remaining six recommendations were removed due to a lack of consensus (Appendix A Table A3). The final guide consists of 67 recommendations categorized according to the six phases of Bailey’s medication self-management model (see Supplementary File S4).

## 4. Discussion

Supporting patients during the process of medication self-management—as a partner in care—is necessary to address challenges in medication management and to empower patients in self-care activities. Hence, in this study, we developed a guide with recommendations for healthcare providers supporting polymedicated patients with medication self-management problems. The recommendations were derived from scientific evidence and existing literature combined with practical experience, knowledge and expertise of experts in the field of medication management of patients with polypharmacy. 

In the first phase of this study, we composed a list of twenty medication self-management problems. Previous research has shown that problems with medication self-management are various and not solely limited to medication intake [6,7,9,10,12,13,14,15,17,18,19,21,22,23,25,42]. Therefore, within the project group, we made a conscious decision not to prioritize just a few problems, but to focus as broadly as possible on the entire process of medication self-management. Bailey’s model of medication self-management provided us with a structure when mapping problems, but also when searching for interventions and actions [4].

We created an overview of interventions or actions that healthcare providers can perform in supporting patients for each medication management problem a patient may experience. For this purpose, a scoping review of systematic reviews, guidelines and grey literature seemed most appropriate [43]. Conducting a systematic review was not convenient, given the multitude of problems does not allow a single systematic search for interventions. Furthermore, only a limited number of interventions have been evaluated in a rigorous clinical trial.

The scoping review resulted in a list of 66 potential actions or interventions, which were formulated as recommendations. However, since multiple medication self-management problems mapped are rather practical in nature (e.g., opening packages, maintaining a daily intake routine), multiple recommendations were not evidence-based but rather experience-based. Therefore, professional experience and expertise were necessary to validate the list of predefined recommendations. Hence, an e-Delphi study was set up for this purpose. The consensus rules along with the rating scales (score 1–4) for this e-Delphi study were inspired by the content validity index, as this simplifies the decision-making process [44]. Furthermore, four-point Likert scales have been shown to produce stable findings in Delphi studies [45]. 

The guidance document was developed with a focus on hospitalized adults with polypharmacy for various reasons. The prevalence of polypharmacy is high and rising [46,47,48]. Furthermore, polypharmacy patients have an increased risk of medication self-management problems as a result of the larger number of medicines prescribed and the increased complexity of the medication regimen [28]. The focus on the hospitalized patient lies in the fact that hospitalization poses risks to patient safety after discharge from the hospital [49,50]. For instance, various changes may have been introduced into the patient’s medication regimen during hospitalization [51,52]. It has been shown that after discharge, 54 to 82% of patients cannot name which medication precisely was changed during hospitalization, and 55% do not use medication at home as initially intended at discharge [53,54,55]. The HARM study showed that 5.6% of all unplanned hospital admissions are medication-related and almost half (46.5%) of these admissions are potentially preventable [56]. Polypharmacy was identified as the most important medication-related potential risk factor for unplanned medication-related hospital admissions [56]. Because of the reasons above, hospitalized patients with polypharmacy are a priority group. Nevertheless, the recommendations may also provide guidance for other patient groups (e.g., non-hospitalized adults or patients taking fewer than five medicines). 

### 4.1. Strengths 

Supporting and guiding patients in medication self-management requires an interdisciplinary approach. A strength of the study, therefore, is the involvement of individuals from different professional groups (physicians, nurses and pharmacists) with relevant experience in clinical practice and/or research regarding medication management. The selection of a heterogeneous expert panel in terms of professional status and work setting might ensure the integration of diverse expertise and experiences which might enhance the relevance of the recommendations for a broader population of patients in different healthcare settings. Not only healthcare providers but also patients were involved during the validation of the recommendations. The panel of patients was rather small, but diverse in terms of socio-demographic characteristics. However, patients’ opinions on the recommendations were consistent, suggesting that the recommendations are meaningful and useful for a variety of patients regardless of their age, level of education or chronic condition. Patients’ opinions were explored simultaneously with the second e-Delphi round. This timing seemed ideal, given that important ambiguities in the recommendations had already been adjusted based on feedback from healthcare providers from the first e-Delphi round. Moreover, during the third round, experts could take patients’ feedback into account. Another strength of the study is the high participation level of experts over the three e-Delphi-rounds. Panelists actively used the free text fields and provided valuable suggestions for the next rounds. 

### 4.2. Limitations

When interpreting the results of this study, there are some limitations to bear in mind. First, given the extensive list of medication self-management problems and the choice to formulate recommendations for each of these problems, only secondary sources (i.e., systematic reviews and guidelines) were included in the scoping review. A search of primary sources (e.g., randomized controlled trials) might have yielded different results. Second, the studies included within the systematic reviews used in the scoping review are usually not rigorous clinical trials. Therefore, the effectiveness of the interventions to support medication self-management (problems) has seldomly been evaluated. Hence, the guide does not include an assessment of the effectiveness or reliability of the recommendations using the Grading of Recommendations, Assessment, Development, and Evaluations (GRADE). The guide provides an overview of the interventions/actions described in the literature, which were in addition assessed as relevant and clear by the expert panel. Third, there is no clear guidance for the level of consensus to be employed in a Delphi study [38,57,58]. Percentage agreement varies widely from 50% to 97% [58], but 75% has been considered to be the minimal level of agreement [38]. We chose to set the cut-off for consensus on relevance and clarity at 80% of expert agreement.

### 4.3. Implications for Practice and Future Research 

The current study resulted in a guidance document with recommendations considered relevant and clear for healthcare providers to support polypharmacy patients with problems in medication management. However, when using the guide in clinical practice, the following aspects should be considered:

1. Supporting patients regarding medication self-management requires an interdisciplinary approach, in which individual healthcare providers can contribute to improving patient medication self-management based on their own competences. In other words, the recommendations can be applied by a variety of healthcare providers (e.g., pharmacists, physicians, nurses) within their areas of expertise, preferably after coordinating with the members of their team.
2. Supporting patients in medication self-management requires the involvement of the patient (and/or informal carer) in the care process. The patient should be explicitly provided with the opportunity to inform the healthcare provider of any problems with self-management, as well as any personal experiences and preferences that might affect medicine use. To arrive at shared decision-making (i.e., decisions that the patient will support), the patient needs to have sufficient information about the problems at hand, the decisions to be made and the options available.
3. Providing advice to patients regarding their medication (and/or its management) should not be limited exclusively to the time of hospital discharge. Patients should receive as much support as necessary in self-management during hospitalization, as well as after discharge.
4. It is not the intention to go through all the recommendations in the guidance document systematically for every patient. The guidance document is intended as a resource for helping healthcare providers intervene when there is evidence of problems in medication self-management. Patients admitted due to drug-related problems (e.g., medication errors, therapy non-adherence) should be regarded as having the highest priority.

This study does not demonstrate whether the developed tool is actually feasible and user-friendly in clinical practice. Therefore, a pilot study is recommended in which the guide would be evaluated at a few hospital wards, starting with an evaluation of the medication self-management problems encountered by patients, followed by healthcare providers supporting these patients during hospitalization with the reported problems based on the guidance document. 

We have to acknowledge that the recommendations stated in the guidance document can become outdated as new evidence is published. Therefore, a periodic reassessment of the guidance document is needed for it to remain valid. According to various authors, an update of recommendations is generally required after three to five years [59,60,61,62].

## 5. Conclusions

Based on a scoping review and e-Delphi study, a guide with recommendations for healthcare providers to support polypharmacy patients with medication self-management problems has been developed. Twenty-three experts reached consensus on the relevance and clarity of 67 recommendations categorized according to the six phases of medication self-management. The guidance document is intended for use by different healthcare providers in an interdisciplinary context to enhance patients’ competences in medication self-management. Future research should focus on the evaluation of the feasibility and user-friendliness of the guide by healthcare providers in clinical practice.

## Figures and Tables

**Table 1:** List of medication self-management problems.

Phase of Medication Self-Management Process (Bailey)	Potential Problems Based on Literature
Picking up the medicines at the pharmacy	1. The patient cannot afford the medicines.
	2. The patient cannot independently pick up medicines at the pharmacy (e.g., due to lack of transport, poor state of health).
	3. The patient has no prescription (or repeat prescription) available/does not know when to obtain a new prescription.
	4. The patient does not know how to obtain new or repeat prescriptions/medicines (e.g., patient does not know whom to contact).
Knowledge and understanding of medicines	5. The patient has no knowledge concerning some or all of the patient’s medicines (i.e., knowledge of name, dosage, timing, administration route and/or indication).
	6. The patient is having trouble understanding information and/or instructions concerning the medicines.
Practical organization and scheduling of medication intake	7. The patient is having trouble organizing/filling a medication box.
	8. The patient is having trouble opening packaging (e.g., removing tablets/capsules from blister packaging).
	9. The patient is having trouble splitting medicines.
	10. The patient is having trouble reading labels/stickers/information/instructions (e.g., on drug packaging, package leaflet, medication schedule).
	11. The patient is having trouble establishing a daily routine.
	12. The patient is having trouble storing medication properly.
Medication intake	13. The patient has swallowing problems.
	14. The patient does not want to take the medication (deliberately).
	15. The patient is taking incorrect doses of medicines (under-dosing, over-dosing, extra doses, forgotten doses).
	16. The patient is not administering the medicines properly.
	17. The patient is taking the medicines at the wrong time of day.
Monitoring therapeutic and adverse effects of medicines	18. The patient lacks knowledge of side effects, contraindications, interactions and/or precautions
	19. The patient lacks knowledge of measures to be taken/does not take appropriate measures in case of side effects or after missing a dose.
Sustaining safe and appropriate medication use	20. The patient has discontinued taking the medication prematurely.

**Table 2:** General characteristics of panelists of the e-Delphi study (n = 23).

Variables	n (%)
**Gender** (female)	14 (60.9)
**Age in years** (median, range)	40 (28–64)
**Country of employment**	
Belgium	13 (56.5)
The Netherlands	10 (43.5)
Professional status *	
Physician	9 (39.1)
Pharmacist	8 (34.8)
Nurse	6 (26.1)
Work setting *	
Clinical practice	19 (82.6)
Hospital care	18 (78.3)
Primary care	2 (8.7)
Residential care	1 (4.3)
Education	11 (47.8)
Policy	2 (8.7)
Research	7 (30.4)

* Participants could indicate more than one professional status/work setting.

**Table 3:** General characteristics of patients (n = 8).

Variables	n (%)
**Gender** (female)	4 (50)
**Age in years** (median, range)	53.5 (23–77)
Caregiver *	2 (25)
**Education**	
Primary education	1 (12.5)
Secondary education	5 (62.5)
Higher education (bachelor)	2 (25)
**Number of chronic medicines** (median, range)	11.5 (5–24)
**Type of chronic condition**	
Cardiac condition	4 (50)
Cystic fibrosis	2 (25)
Malignancy	2 (25)

* Two participants were not only a patient themselves, but they were also an informal carer for a family member and prepared medication for him or her.

## Data Availability

Data are available from the corresponding author upon reasonable request.

## Supplementary Materials

The following supporting information can be downloaded at https://www.mdpi.com/article/10.3390/healthcare11111545/s1: Supplementary File S1: Excel file with the content of the first e-Delphi round, as was presented to the experts (English translation); Supplementary File S2: Survey used in patient panel (English translation); Supplementary File S3: Summary of the included records in the scoping review; Supplementary File S4: Guidance document with recommendations (English translation).

## Appendix A

**Table A1:** Search terms.

Concept	Search Term
Polypharmacy/medication	‘polypharmacy (MeSH)’, ‘polypharmacy’, ‘polymedication’, ‘multiple drug therapy’, ‘multidrug therapy’, ‘multidrug regimen’, ‘multiple medication’, ‘multiple drug*’, ‘medication’, ‘medicine*’, ‘drug*’
Self-management	‘self manag*’, ‘self-management’, ‘self-management (MeSH)’, ‘self-management support’
Filling a prescription	‘obtain’, ‘purchase’, ‘access*’, ‘prescription’
Knowledge and understanding of medication	‘medication knowledge’, ‘drug knowledge’, Patient Medication Knowledge (MeSH), ‘Patient drug knowledge’, ‘knowledge’, ‘comprehension’, comprehension (MeSH), ‘dosage’, ‘dose’, ‘administration route’, ‘indication’, ‘readability’, ‘comprehension’, ‘labels’, ‘information’, ‘instruction’,
Medication intake	‘dysphagia’, ‘presbyphagia’, ‘swallowing problems’, ‘swallowing difficulties’, ‘swallowing disorder’, deglutition disorders (MeSH)
Monitoring therapeutic and adverse effects	‘adverse effect*’, Drug-related side effects and adverse effects (MeSH), ‘therapeutic effect’, ‘precaution’, ‘side effect’, ‘contraindication*’, ‘contra-indication*’, contraindications (MeSH), ‘interaction’, Drug Interactions (MeSH), ‘warning*’, ‘monitoring’
Sustaining appropriate medication use	‘persistence’, ‘medication persistence’, ‘medication adherence (MeSH)’, ‘medication adherence’, ‘drug adherence’, ‘medication compliance’, ‘Medication Nonadherence’, ‘Medication Non-Adherence’, ‘Medication Non Adherence’, ‘Medication Noncompliance’, ‘Medication Non-Compliance’, ‘Medication Non Compliance’
Population	‘patient*’, ‘client*’, ‘consumer*’, ‘adult’, adult (MeSH)

**Table A2:** Summary of grouped recommendations by phase of medication self-management.

	Number of Recommendations in Each Phase			Percentage of Statements Where Consensus Was Achieved for Both Relevance and Clarity (n) *		
Phase of Medication Self-Management	Round 1	Round 2	Round 3	Round 1	Round 2	Round 3
Picking up the medicines at the pharmacy	7	8	10	0% (0)	25% (2)	80% (8)
Knowledge and understanding of medication	13	12	13	23% (3)	75% (9)	92% (12)
Organizing and planning medication use	13	18	18	54% (7)	94% (17)	100% (18)
Medication intake	22	20	20	55% (12)	90% (18)	95% (19)
Monitoring therapeutic and adverse effects	9	11	11	78% (7)	73% (8)	82% (9)
Sustaining safe and appropriate medication use through the duration of the prescription	2	2	2	0% (0)	100% (2)	100% (2)
**Total** (**across all phases**)	66	71	74	44% (29)	79% (56)	92% (68)

* Consensus on relevance and clarity was achieved when ≥80% of the experts assigned a score of 3 (relevant/clear) or 4 (very relevant/very clear) to an item **OR** when ≥80% of the experts assigned a score of 1 (very irrelevant/very unclear) or 2 (irrelevant/unclear) to an item.

**Table A3:** Number of changes to recommendations in each round according to type.

	Number of Changes after Round 1						Number of Changes after Round 2						Number of Changes after Round 3					
Phase of Medication Self-Management	Rephrased	Adapted to Context	Combination of Recommendations	Splitting up Recommendations	New Recommendation Added	Removed due to Overlap	Rephrased	Adapted to Context	Combination of Recommendations	Splitting up Recommendations	New Recommendation Added	Removed due to Consensus on Irrelevance	Rephrased	Adapted to Context	Combination of Recommendations	Splitting up Recommendations	New Recommendation Added	Removed *
Picking up the medicines at the pharmacy	5	2	4	-	3	-	3	1	-	2	-	-	1	-	-	-	-	2
Knowledge and understanding of medication	6	1	6	-	3	-	4	1	-	1	-	-	-	-	-	-	-	1
Organizing and planning medication use	6	-	-	-	4	-	3	-	-	-	-	-	-	-	-	-	-	1
Medication intake	6	-	1	-	1	2	1	-	-	-	-	1	-	-	-	-	-	1
Monitoring therapeutic and adverse effects	2	-	-	-	2	-	3	-	-	-	-	-	-	-	-	-	-	2
Sustaining safe and appropriate medication use through the duration of the prescription	1	-	2	-	1	-	-	-	-	-	-	-	-	-	-	-	-	-
**Total** (**across all phases**)	26	3	13	-	14	2	14	2	-	3	-	1	1	-	-	-	-	7

* In the phase of medication intake, one recommendation was removed since there was consensus on irrelevancy. The recommendations in the other domains were removed due to a lack of consensus after the third e-Delphi round.
