# Supplementary material for: The Development of Recommendations for Healthcare Providers to Support Patients Experiencing Medication Self-Management Problems
Source: Healthcare (Basel). 2023 May 25;11(11):1545. doi: 10.3390/healthcare11111545 (PMC10253050; doi:10.3390/healthcare11111545)
Supplement: Supplementary file 1 [file healthcare-11-01545-s001.zip › Supplementary_file_S1-Expert_survey_Delphi_round_1.pdf]

Recommendations for healthcare providers to support polypharmacy patients with medication self-management problems: a Delphi-study

Instructions Delphi study

You received this Excel file together with a guidance document with recommendations for healthcare providers to support patients with polypharmacy in case of problems with medication self-management. Both documents should be used together. Please read through the instructions below and complete each tab afterwards.

By completing this document, you agree to participate in this study. Participation is entirely voluntary.

Tab 2 'Personal data'
Please enter your personal information and/or select an answer. This data will be used to describe the composition of the expert panel.

Tab 3 'Evaluation background information'
In this tab, various titles and subtitles are displayed. These titles and subtitles correspond to the different parts of the guidance document with recommendations. Please evaluate the different parts of the guidance docuement according to two criteria:
1. Relevance of the content for users in practice
2. Clarity of the wording of the content

For each evaluation criterion, give a score from 1 to 4 (see table 1). The cells are pre-set, so that no incorrect values can be entered. The cells will be automatically coloured according to the score entered. Space is provided in the last column for comments or suggestions.

Tab 4 'Evaluation recommendations'
The first column shows the different phases of Bailey's medication self-management model. In the second column, you will see different categories of medication self-management problems within each phase. Recommendations were formulated within each of these categories. In the third column, you will find the number of the recommendation. This number corresponds to the number in the guidance document.

Please evaluate each recommendation using two criteria:
1. Relevance of the recommendation to practice/healthcare providers (useful, feasible, effective?).
2. Clarity of the wording of the recommendation

For each evaluation criterion, give a score from 1 to 4 (see table 1). The cells are pre-set so that no incorrect values can be entered. The cells will be automatically coloured according to the score entered. If you feel that a recommendation does not belong to your area of expertise, you can tick it in the relevant column. If you wish, you can add comments or make suggestions to make adjustments to existing recommendations. In the last column, space is provided to formulate additional recommendations within a category of medication self-management problems, based on your experience as an expert.

Tab 5 'Evaluation format'
With this section, we want to identify the most appropriate format of the guidance document to implement the recommendations into practice. Please evaluate the current format of the guidance and other suggestions using the following criteria:
1. User-friendliness in practice
2. Feasibility in practice

For each evaluation criterion, give a score from 1 to 4 (see table 2). The cells are pre-set so that no incorrect values can be entered. The cells will be automatically coloured according to the score entered. In the last column, space is provided for comments or suggestions.

Questions?
Please do not hesitate to contact us with any questions.
Thank you very much in advance for your participation in this study. Your contribution is very important to establish appropriate practical recommendations to support patients with medication self-management problems.

Contact details
Laura Mortelmans, *laura.mortelmans@uantwerpen.be*
Prof. dr. Tinne Dilles, *tinne.dilles@uantwerpen.be*

TABLE 1

	Relevance	Clarity
Score 1	Completely irrelevant	Very unclear
Score 2	Irrelevant	Unclear
Score 3	Relevant	Clear
Score 4	Very relevant	Very clear

TABLE 2

	User-friendliness	Feasibility
Score 1	Not user-friendly at all	Not feasible at all
Score 2	Not user-friendly	Not feasible
Score 3	User-friendly	Feasible
Score 4	Very user-friendly	Very feasible

Code number: ....

**Personal data**<br><br>*Please complete/select the answers to the following questions.*		
**What is your age?**	*Please insert your age here.*	
**What is your gender?**	☐	Man
	☐	Woman
**Which country do you work in?**	☐	Belgium
	☐	The Netherlands
	☐	Other country: ....
**In which setting are you working?**<br>(Multiple answers possible)	☐	Clinical practice
		☐ Hospital care
		☐ Primary care
		☐ Residential care
	☐	University college/university
	☐	Policy
	☐	Research
	☐	Other setting: ....
**What is your professional status?**<br>(Multiple answers possible)	☐	Physician
	☐	Pharmacist
	☐	Nurse
	☐	Other: ....
**Within which domain(s) do you have expertise?**<br>(Please complete)		....
		....
		....
		....
**Would you like to be recognised by name as an expert in the acknowledgements of corresponding publications?**	☐	Yes
	☐	No

Title (according to guidance document)	Subtitle (according to guidance document)	Relevance (1 = completely irrelevant, 4 = very relevant)	Clarity (1 = very unclear, 4 = very clear)	Add comments or suggestions here, if desired.
1. Introduction/background	1.1. Medication self-management			
	1.2 Factors contributing to medication self-management problems			
	1.3 Impact medication self-management problems			
	1.4 Rationale			
2. Objective				
3. End users				
4. Target patient population				
5. Methodology	5.1STEP 1: Mapping problems relating to medication self-management			
	5.2 STEP 2: Development of the guidance document			
	5.3 STEP 3: Validation of the guidance document			
	5.4 STEP 4: Evaluation in practice			

Phase medication self-management process (according to Bailey's model)	Subcategory medication self-management problems	Number recommendation	Relevance (1 = completely irrelevant, 4 = very relevant)	Clarity (1 = very unclear, 4 = very clear)	Is not in my area of expertise	If desired, add comments or suggestions for adjustments to existing recommendations.	If desired, add additional, new recommendations within a subcategory of medication self-management problems.
6.1 Picking up the medicines at the pharmacy	6.1.1 The patient experiences financial problems	6.1.1.1			☐		
		6.1.1.2			☐		
	6.1.2 Patient has no or insufficient medication supply	6.1.2.1			☐		
		6.1.2.2			☐		
		6.1.2.3			☐		
		6.1.2.4			☐		
		6.1.2.5			☐		
6.2 Knowledge and understanding of medication	6.2.1. The patient lacks knowledge of the medicines: name, indication, dosage, time of administration, method of administration	6.2.1.1			☐		
		6.2.1.2			☐		
		6.2.1.3			☐		
		6.2.1.4			☐		
		6.2.1.5			☐		
	6.2.2. The patient is having trouble understanding information and/or instructions concerning the medicines.	6.2.2.1			☐		
		6.2.2.2			☐		
		6.2.2.3			☐		
		6.2.2.4			☐		
		6.2.2.5			☐		
		6.2.2.6			☐		
		6.2.2.7			☐		
		6.2.2.8			☐		
6.3 Organizing and planning medication use	6.3.1. The patient is having trouble preparing the medication	6.3.1.1			☐		
		6.3.1.2			☐		
		6.3.1.3			☐		
		6.3.1.4			☐		
		6.3.1.5			☐		
		6.3.1.6			☐		
	6.3.2. The patient is having trouble reading labels, package leaflets, information, instruction	6.3.2.1			☐		
		6.3.2.2			☐		
	6.3.3. The patient is having trouble establishing a daily routine	6.3.3.1			☐		
		6.3.3.2			☐		
	6.3.4. The patient is having	6.3.4.1			☐		

	[illegible] trouble storing the medicines properly	6.3.4.2			☐		
		6.3.4.3			☐		
6.4 Medication intake	6.4.1. The patient has swallowing problems	6.4.1.1			☐		
		6.4.1.2			☐		
		6.4.1.3			☐		
		6.4.1.4			☐		
		6.4.1.5			☐		
		6.4.1.6			☐		
	6.4.2 The patient is not using the medicines properly	6.4.2.1			☐		
		6.4.2.2			☐		
		6.4.2.3			☐		
		6.4.2.4			☐		
		6.4.2.5			☐		
		6.4.2.6			☐		
		6.4.2.7			☐		
		6.4.2.8			☐		
		6.4.2.9			☐		
		6.4.2.10			☐		
	6.4.3. The patient does not want to take the medicines	6.4.3.1			☐		
		6.4.3.2			☐		
		6.4.3.3			☐		
		6.4.3.4			☐		
		6.4.3.5			☐		
		6.4.3.6			☐		
6.5 Monitoring therapeutic and adverse effects	6.5.1. The patient lacks knowledge about the desired and adverse effects (side effects and interactions) of the medicines	6.5.1.1			☐		
		6.5.1.2			☐		
		6.5.1.3			☐		
	6.5.2. The patient is having trouble taking appropriate measures when side effects occur	6.5.2.1			☐		
		6.5.2.2			☐		
		6.5.2.3			☐		
		6.5.2.4			☐		
		6.5.2.5			☐		
		6.5.2.6			☐		

6.6 Sustaining safe and appropriate medication use	6.6.1. The patient has discontinued taking the medication prematurely	6.6.1.1			☐		
		6.6.1.2			☐		

	User-friendliness (1= not user-friendly at all, 4= very user-friendly)	Feasability (1 = not feasible at all, 4= very feasible)	If desired, add comments or new suggestions on the format in which the recommendations should be offered in practice.
Current format of the guidance document			
Table of contents with crosslinks to each section/medication self-management problem			
Recommendations in tabular form, structured by medication self-management problem			
Index cards per medication self-management problem			
Online tool: (free) multi-page website			
