# Supplementary material for: The Development of Recommendations for Healthcare Providers to Support Patients Experiencing Medication Self-Management Problems
Source: Healthcare (Basel). 2023 May 25;11(11):1545. doi: 10.3390/healthcare11111545 (PMC10253050; doi:10.3390/healthcare11111545)
Supplement: Supplementary file 1 [file healthcare-11-01545-s001.zip › Supplementary_file_S2-Patient_survey.pdf]

Universiteit Antwerpen

Study "Recommendations for healthcare providers to support polypharmacy patients with medication self-management problems"

1. General data

This information is only used to describe the type of patients who have evaluated the guidance document.

1.1. What is your gender? Please tick.

☐ Man

☐ Woman

☐ I can't say

1.2. What is your age ………………………………… year

1.3. What is your highest level of education? Please tick.

☐ None

☐ Primary education

☐ Secondary education

☐ Higher vocational education (A2/HBO5)

☐ University college (A1/bachelor)

☐ University (licentiate/master/doctorate)

☐ Other: …………………………………………………………..

1.4. How many different medicines do you take daily at home?

………………………………. medicines

1.5. How many different medicines have you been taking at home for more than three months?

………………………………. medicines

1.6. To what extent do you take your medication independently at home?

☐ I prepare and administer my medication completely independently.

☐ I receive help with preparation (e.g. filling medication box), but administer them independently.

☐ I receive help with preparation (e.g. having my medication box replenished) and administration (e.g. medication is administered by family, (home) nurse or informal carer)

2. Review of the guidance document with recommendations

The guidance document has been developed for use by healthcare providers. They can use the recommendations to support patients in taking their medicines. Because the guidance document can have an impact on patient care, we also like to include patients' opinions.

Please read through the guidance document with advice for healthcare providers. You only have to look at the section '6. Practical recommendations' (pages 12 to 23).

- Mark the recommendations you find useful in **green**. You may also mark only the number of the recommendation.
- Mark the recommendations you do not find useful in **pink**. You may also mark only the number of the recommendation.
- If you have no opinion on the usefulness of a particular advice, do not mark this advice.

After reading the guidance document, answer the questions below.

2.1. What do you think in general about the guidance document? Please tick the extent to which you agree with the statements below.

	Agree	Rather agree	Neutral	Rather disagree	Disagree
a) In general, the guidance document is useful.					
b) The recommendations in the guidance document are useful for healthcare providers to help patients who experience problems with their medicines.					
c) If I experienced problems with my medicines myself, healthcare providers could help me with the recommendations from the guidance document.					
d) The guidance document is important so that healthcare providers can properly advise and support patients if they experience problems with their medication.					

2.2. Is there any recommendation from the guidance document that you do not find useful? Please tick.

☐ Yes, there are recommendations that I do not find useful (go to question 2.3).
☐ No, all recommendations from the guidance document are useful (go to question 2.4).

2.3. Which recommendations from the guidance document do you find not useful? Please write down the number of the recommendation from the guide and indicate why you think the advice is not useful.

**Number recommendation**	**Reason why you do not find the recommendation useful.**

2.4. Are there any recommendations regarding medication management at home that you think is missing in the guidance document? Please tick.

☐ Yes, recommendation are missing in the guidance document (go to question 2.5).
☐ No, no recommendations are missing in the guidance document (go to question 2.6).

2.5. What recommendations regarding medication management at home do you think are missing in the guidance document?

– ………

………

………

………

– ………

………

………

………

– ………

………

………

………

– ………

………

………

………

2.6. Do you have any other comments on the guidance document? Please tick.

☐ No
☐ Yes:

………

………

………

………

………

………

………

………

………

2.7. How difficult was it for you to understand the recommendations in the guidance document ? Please tick.

Very easy	Easy	Neutral	Difficult	Very difficult

Thank you for your time and participation!
