# Supplementary material for: The Development of Recommendations for Healthcare Providers to Support Patients Experiencing Medication Self-Management Problems
Source: Healthcare (Basel). 2023 May 25;11(11):1545. doi: 10.3390/healthcare11111545 (PMC10253050; doi:10.3390/healthcare11111545)
Supplement: Supplementary file 1 [file healthcare-11-01545-s001.zip › Supplementary_File_S3_Summary-included-records.pdf]

Table S2: Summary of the included records in the scoping review (n= 36)

	Author, year [reference]	Country	Objective(s)	Design/ record type	(Target) setting	(Target) patient population	Outcomes	Main findings (intervention/ action/ recommendation described)
**1.**	Bailey et al., 2015 [1]	USA	To examine, summarize, and update best practices for conveying written prescription medication information and instructions.	Systematic review: 31 studies included<br>- 10 experimental studies with randomization<br>- 21 studies used cross-sectional, observational, or qualitative methodologies	Ambulatory care	All patients in ambulatory care	Patient preferences, acceptability, and comprehension (correct interpretation of content including risk or demonstrated use of a medication)	- Studies examining patient preferences and acceptability of labelling strategies, as well as those exploring patient comprehension and recall of drug instructions, found clear benefits in the use of (1) plain language and (2) more intuitive, patient-centered organization and formatting.<br>- Use lists, headers, and white space to promote readability as well as to organize content according to patient schemas<br>- Avoid the use of medical jargon or challenging words or phrases<br>- The use of more explicit dosing instructions as exemplified in the Universal Medication Schedule time frames , which ties medication use to specific time periods (morning, noon, evening, bedtime), significantly improved patient understanding and demonstrated use of medications.<br>- The inclusion of patient-vetted icons to support attention to and comprehension of instructional or warning information had conflicting findings.
**2.**	Belgische Vereniging voor Gerontologie en Geriatrie, 2020 [2]	Belgium	Informing patients/informal caregivers, as well as primary care providers (GP, family pharmacist, home nurse), about discharge medication to ensure good transmural pharmaceutical care, reduce the risk of drug-related problems and optimise adherence.	Guideline	Hospital (hospital ward or outpatient department)	Older patients	Not applicable	- Find out to what extent the patient himself is responsible for medication intake, or whether the medication is prepared and/or administered by an informal carer.<br>- Check what information regarding discharge medication the patient/informal carer has already received and try to evaluate what the patient/carer knows.<br>- Establish an up-to-date medication schedule of discharge medication.<br>- The medication schedule at discharge should include at least the following for each medicine: brand name (including dose, dose unit and formulation), indication, dose frequency (per day, per week), time of administration (+ intake in relation to diet if important), quantity + unit per administration, route of administration, indicate stopping date for medicines with a fixed duration (e.g. antimicrobials, pain medication)<br>- Describe or indicate all medication changes that occurred during the hospital stay (what was stopped, started or changed) and, if possible, the reason(s) for

								these changes: for example, by preparing a pharmaceutical discharge letter and/or by using colours to visually indicate what is new and what has changed on the medication schedule.<br>- Record all discontinued medicines (do not list them in the medication schedule itself, but separately to avoid confusion).<br>- Use the medication schedule as a guide during the counselling session with the patient/carer. This chart should be clear and include only terms that the patient/carer understands. Clarify where necessary.<br>- Explain all medication changes (what is new, what was changed and what was stopped) and if possible the underlying reason(s) when you assess that this is important to communicate to the patient/carer.<br>- Discuss the following items for all newly started medicines and for all medicines for which additional information is appropriate (e.g., High Risk Medication):<br>o Information on the indication, dosing frequency together with amount and unit per administration, administration time and -route. If necessary, give additional instruction (e.g. correct inhalation technique, administration of eye drops,….<br>o Provide information on the most frequent possible side effects: how to avoid them, how to recognise them, what to do if they occur, when to contact a healthcare provider.<br>o Provide information on drug-specific requirements (e.g. reimbursement by certificate, only available in hospital pharmacy).<br>- Emphasise the importance of adherence and the possible consequences of non-adherence: Try to optimise adherence by identifying potential problems and suggesting patient-specific solutions (e.g. additional education, optimisation of the medication schedule, tools such as pillbox or reminder SMS)<br>- Indicate to contact the GP or family pharmacist to know what to do after a forgotten medicine intake.<br>- Provide sufficient medication supply to cover the period between hospital discharge and visit to office pharmacy: Inform the patient/carer about how many days' medication was given for. If applicable, indicate

								which home medication the formulary product (if any) corresponds to. formulary product corresponds to. Inform the patient/carer which medications have already been administered on day of discharge. Provide information on the appropriate storage temperature.<br>- Ask for feedback: check whether the patient/carer finds the discharge medication schedule clear and complete.<br>- Check that the patient/carer has understood everything correctly using the 'teach-back' method, especially for newly started medication.
**3.**	Brega et al., 2015 [3]	USA	To provide evidence-based guidance to support primary care practices in addressing health literacy. The Toolkit can help practices reduce the complexity of health care, increase patient understanding of health information, and enhance support for patients of all literacy levels.	Toolkit report	Primary care and community clinics	Patients with limited health literacy	Not applicable	The toolkit comprises 21 tools addressing 4 domains that are important to promote health literacy: spoken communication, written communication, Self-Management and Empowerment, Supportive systems. Detailed information on the tools can be found in the toolkit. Some examples of recommendations are listed below.<br><br>- Use Plain, Non-medical Language<br>- Focus on “need-to-know” and “need-to-do”<br>- Show how it’s done: demonstrate how to take medicines<br>- Use Teach-Back Method to confirm patient understanding. Use questions such as *“Tell me what you've understood about this medicine?" or "I want to make sure I have explained your medicine clearly, can you tell me how you think you will take this medicine?"*<br>- Use Graphics<br>- Encourage questions
**4.**	Capiau et al., 2020 [4]	Belgium	(1) To provide an overview of reported components of medication counselling in older patients (aged ≥ 65 years) prior to hospital discharge and (2) to review the effectiveness on reported clinical outcomes such as hospital readmissions, medication adherence medication knowledge and ED visits	Systematic review: 29 studies included<br>- 16 randomized controlled trials<br>- 13 non-randomized studies	Hospital discharge setting	Older patients	Hospital readmissions, medication adherence, medication knowledge, ED visits	Components of counselling interventions:<br>- Discussing the dose and dosage of patients’ medications (19/29; 65.5%)<br>- Providing a paper-based medication list” (19/29; 65.5%)<br>- Explanation of the indications of the prescribed medications (17/29; 58.6%).<br>- Potential adverse drug reactions that patients might experience during therapy (12/29; 41.4%)<br>- Information about medications stopped, newly started drugs and drugs that were changed (e.g. altered dose or frequency) (8/29; 27.6%).<br>- Importance of medication adherence (6/29; 20.7%)

								- Information about the storage of medications, instructions on how to deal with missed doses, dietary and lifestyle education, information about the benefits of therapy, the cost of therapy and explanation of therapeutic goals.<br>- Use of teach-back method to ensure patients understood the instructions provided (5/29, 17.2%)<br>Impact of discharge counselling on clinical outcomes:<br>- Significant impact on medication knowledge (in 7/8 studies)<br>- Significant impact on medication adherence (in 9/12 studies)<br>- Significant impact on hospital readmissions (in 5/14 studies) and ED visits (in 2/6 studies).
**5.**	Conn et al., 2016 [5]	USA	To synthesize findings from medication adherence intervention studies conducted among adults with medication adherence difficulties.	Systematic review: 53 primary intervention studies included	Not specified	Adults with medication adherence problems	Medication adherence	- Studies that employed prompts or cues for taking medications had larger effect sizes than studies that did not (0.497 vs. 0.234, p = .034). Typical prompts might include cell phone alarm reminders, locating medications in a particular location to cue medication taking such as on the kitchen table for medication to be consumed with meals, or placing reminders in strategic locations such as a note on the bathroom mirror.<br>- Habit-focused interventions in which participants' daily habits were linked to taking medications were also effective in increasing medication adherence relative to interventions lacking this component (0.574 vs. 0.222, p = .007)
**6.**	De Bodt et al., 2015 [6]		To provide healthcare providers with a resource to provide adequate dysphagia care.	Book	Not specified	Patients with dysphagia	Not applicable	- Advise patients to swallow with the chin on the chest (chin-tuck technique)<br>- Dysphagia patients may have difficulty taking medication via the oral administration route. Consider other administration forms of medication.<br>- A speech therapist can provide swallowing therapy.
**7.**	Dietrich et al., 2020 [7]	Switzerland	To investigate any type of benefits associated with medication charts provided at transition points.	Systematic review: 30 studies included<br>- 4 randomized controlled trials<br>- 10 cohort studies<br>- 7 cross-sectional studies<br>- 2 qualitative studies<br>- 4 surveys<br>- 2 reports	Ambulatory care, primary care, hospital care, residential care	General population	Knowledge, safety, communication between patient and healthcare provider, empowerment, interdisciplinary cooperation	- Patients with access to a medication chart had a better knowledge of their medication that concerned knowledge of indication, medication name, dosage and dosage frequency<br>- A medication chart was identified as one of the parameters to ensure medication safety as well as patient safety: a medication chart helped patients to avoid making mistakes with medication (i.e., wrong time, wrong pill, missed or delayed doses).<br>- It is essential to inform the patient about how to use the medication chart and why it is needed.

				- 1 systematic review				- A medication chart has to be accurate, up-to-date and comprehensive to achieve the benefits.<br>- The documentation of patients' medication is crucial at any transition of care (e.g., entry or discharge from hospital) and at any change of treatment (prescribing or deprescribing) to avoid medication errors, and eventually improve and ensure safety.
**8.**	Farmaka, 2021 [8]	Belgium	To promote responsible medication use	Web Page	Not specified	Older persons	Not applicable	Recommendations for splitting medicines:<br>- Check whether the tablet contains a break line allowing division into the desired dose. In the absence of a break line, do not divide, unless expressly stated otherwise in the package leaflet.<br>- Check whether the form is a modified-release formulation. If so, do not divide the tablet unless expressly stated otherwise in the package leaflet.<br>- Ensure that the medicine is correctly divided and that it is not crumbled. If necessary, use a tablet divider and follow the instructions for use.<br>- Pay particular attention to medicinal products with a narrow therapeutic range.<br>- Take care that the divided medicine is stored optimally.<br>Recommendations for crushing medicines and administration of crushed medication:<br>- Always check carefully whether the medicine qualifies for crushing and whether there is another more appropriate form of administration (e.g. a liquid form). If necessary, consult https://vza.be/bibliotheek-pletmedicatie/pletfiches<br>- Use a tablet crusher and follow the instructions for use.<br>- To mask the bad taste, crushed medication is sometimes administered with something other than water. To avoid complexation, it is best not to mix the medication with calcium-containing food (e.g. yoghurt, milk) but to use jam, for example. This recommendation is based on experience. Caution! A number of medicines should not be taken with food (e.g. voriconazole). Consult https://vza.be/bibliotheek-pletmedicatie/pletfiches if necessary.<br>- After crushing, use the mixture immediately to minimise as many stability problems as possible.<br>- Ensure that the full dose is taken (recipient rinses well).

								- Ensure patients do not mix up or share food or drink (case report of elderly people who regularly tasted each other's pudding (mixed with drug) and eventually ended up in A&E).<br>- Avoid consuming hot drinks.<br>- Crushing medicines may be useful to facilitate ingestion in patients with swallowing difficulties.
**9.**	Farmaka, 2017 [9]	Belgium	To promote responsible medication use	Web Page	Not specified	Older persons	Not applicable	- Advise patients to swallow with the chin on the chest (chin-tuck technique)
**10.**	Federaal agentschap voor geneesmiddelen en gezondheids-producten [10]	Belgium	To inform patients on proper storage of medicines	Web Page	Not specified	Patients in general	Not applicable	**Storing medicines**<br>- There is always an expiry date on the packaging of a medicine. On the packaging, the expiry date is indicated by the letters 'EX', followed by the words month/year or day/month/year. Once this date has passed, you should no longer use the medicine.<br>- Eye drops should not be stored for more than one month once the bottle has been opened. Other medicines with a limited storage period are: some antibiotics in syrup form after dissolving, medicines prepared by the pharmacist, some medicines supplied by the pharmacist as solutions or suspensions. Read carefully the section 'How to store' in the package leaflet of the medicine.<br>- To maintain the effectiveness of your medicine, always store it in the right conditions. Under the influence of heat, for example, a medicine may become less effective or even toxic. This is why the packaging and package leaflet of each medicine state storage instructions and also the temperature at which you should store the medicine. Follow these instructions!<br>- Try to keep your home pharmacy up-to-date. Sort your medicines regularly and take out expired ones. You can hand in expired medicines to your pharmacy for destruction.
**11.**	Ha Dinh et al., 2016 [11]	Australia	To identify the effectiveness of teach-back education on disease-specific knowledge, medication and care adherence, and specific self-management skills in adult patients with chronic diseases.	Systematic review: 10 studies included<br>- 8 non-randomized/ randomized controlled trials<br>- 1 cohort study<br>- 1 before-after study	Any healthcare setting	Adults aged 18 years and over with one or more than one chronic disease	Adherence, self-management, disease-specific knowledge, readmission, knowledge retention, self-efficacy and quality of life	- Only 1 RCT reported adherence as a measured outcome. The teach-back method improved medication adherence significantly.<br>- Four out of five studies reported significant increase in knowledge scores following the teach-back intervention.<br>- Inconclusive results on the effect of the teach-back method on self-efficacy.<br>- No effect of the teach-back method on quality of life found.

								- The teach-method reduced readmission rates and hospitalization, but results were not always statistically significant.<br><br>Recommendations stated by Ha Dinh et al.<br>- Integrate the teach-back method into education for patients and prioritize disadvantaged people such as those with chronic diseases, low literacy, cognitive impairment and older adults. (Grade A)<br>- Involve all health care professionals in using the teach-back method to maximize patients' understanding of disease state, treatment, care and prevention of complications. (Grade A)<br>- Use the teach-back method in follow-up and reminding patients to maintain the obtained knowledge, adherence and self-efficacy. (Grade A)
**12.**	Instituut Verantwoord Medicijngebruik [12]	The Netherlands	BEM tools are used to identify where any medication management problems are occurring and to monitor whether the client's management of medication is still justified.	Web Page	Long-term (elderly) care	Older people	Not applicable	**Tips regarding medication management**<br>- The pharmacy is responsible for a readable and up-to-date overview of the medication, which is updated with every medication change. Together with the client, the pharmacy must check that the medication overview is complete and correct (including self-care medication). Hence, there is a 'verified current medication overview'.<br>- It is helpful if the client writes down the name, address details and telephone number of the GP and pharmacy and keeps them in an obvious place. Program a (easy-to-use) telephone with memory function with important numbers of GP, pharmacy, hospital, 112.<br>- Have the client keep track on a calendar or diary of when repeat medication is needed. If this does not work, you can involve the informal carer. Advise the client to order the medication in time.<br>- Sometimes a new pair of glasses is needed. You can also ask the pharmacy if it is possible to print names, dosages and instructions for use of medicines more legibly on the packaging.<br>- Encourage the client to contact the GP or pharmacy if the medicines do not work, if there are side effects of there is something wrong with the medicines.<br>**Tips for medicine use**<br>- Difficulties with opening packages: The client can use an aid - such as 'Open Aid' - for opening and closing vials. This can also be used to open the foil of certain vials. Opening foil can often also be done with

								ordinary (household) openers for bottles and jars. To push through tablets or capsules from a blister pack, the client can purchase a tablet push-through device or 'Pill Out'. This tool is available from pharmacies or via the internet.<br>- Taking medicines at the wrong time: Write on a calendar the days of (irregular) use. If necessary, the informal carer can have a task in helping to remember. The GP or pharmacist can set a rhythm that is easy to remember or try to limit irregular medicine use. Discuss this with the client/informal caregiver or GP (after client consent). Provide education if the client does not understand the reason for different intake times.<br>- It is clearly stated on the packaging if a medicine has a limited shelf life after opening. Check whether the client uses such medicines. These are mainly drops, drinks and some creams or ointments. When opening, put the date of opening and/or the expiry date on the packaging with a waterproof marker.<br>- Review with the client what can help to make specific use instructions a regular habit. For example: “take on an empty stomach”: place the medicine next to the bed in the evening with a glass of water<br>- Advise the client to swallow with the chin on the chest.<br>○ Replacing tablets with a drink may solve swallowing problems. Consult your GP or pharmacist the possibilities.<br>○ Taking medicines with custard or applesauce, for example, can make swallowing easier. Check with the pharmacy whether the combination is possible.<br>○ Thickening a drink with a thickener can make swallowing easier. Ask the pharmacist about the possibilities**.**<br>○ Never crush medicines without instructions on the administration list and consult with the doctor or pharmacist. There is an instructional video on swallowing medicine on apotheek.nl<br>- A tablet splitter can help split tablets that do not have a break line.<br>- If you have problems opening the medicine package, ask the pharmacist if the medication is also available in a packaging that is easy to open.

**13.**	Koninklijke Nederlandse Maatschappij ter bevordering der Pharmacie [13]	The Netherlands	To provide medicine users with accurate, up-to-date, understandable and practical medicine information.	Website (Aptoheek.nl)	All medicine users (setting not specified)	All medicine users	Not applicable	- An online reading tool provides support for medicine users who have reading difficulties.<br>- Using medicines correctly is only possible if the medicine user have understood the instructions properly. Apotheek.nl offers clear instruction videos in four languages (Dutch, English, Turkish and Arabic).<br>- Apotheek.nl offers short videos with explanations by pharmacists, for patients with reading difficulties or patients who have difficulty understanding medicine information.<br>- Apotheek.nl offers medicine explanations in sign language.<br>- Apotheek.nl offers videos explaining the terms on the medicine pack.<br>- Spoken and written texts are easier to understand if they are accompanied by an image. That is why there are icons in the medicine videos and on the medicine pages. The icons show what the medicine is used for (such as: 'for the heart'), how it should be used (such as: 'take with food'), and what the most important warnings and side effects are. The icons support the patient in his or her medicine use.
**14.**	Koninklijke Nederlandse Maatschappij ter bevordering der Pharmacie, 2019 [14]	The Netherlands	To transfer medication data in the patient's network so that prescribers, pharmacists, and administrators can provide continuity of care at the time of prescribing, dispensing and administering within the chain and make responsible risk assessments for safe medical and pharmaceutical care.	Guideline	The guideline applies to any situation in which medication is prescribed, changed or stopped and to any situation in which pharmaceutical care is provided and medication is handed or administered.	The guideline applies to any situation in which medication is prescribed, changed or stopped and to any situation in which pharmaceutical care is provided and medication is handed or administered.	Not applicable	The basic medication data set contains at least the following:<br>(1) Agreements per medicine: drug, commercial product name and generic name; dosage (frequency, dose and dosage form) and therapeutic duration of use, time of intake, stop and end date (so chronic use is recognisable); route of administration; strength per administration route<br>(2) Stopped or changed medication in the last two months<br>(3) Patient details<br>(4) Abnormal renal function values + date of determination<br>(5) Drug hypersensitivities (intolerances, allergies and serious adverse reactions and patient-relevant contraindications)<br>(6) Reason(s) for prescription, at least for the 23 legally required medications that have multiple indications in various dosages and a narrow therapeutic range or risk of serious adverse effects; and side effects;<br>(7) Reason(s) for stopping and changing medicines<br>(8) Date when changes in medication were made.

**15.**	Maghroudi et al., 2021 [15]	The Netherlands	To summarize the available research findings on which textual elements facilitate or hinder the correct interpretation of drug label instructions in relation to patients' health literacy.	Systematic review: 28 studies included<br>- 19 qualitative studies with interviews<br>- 4 survey studies<br>- 3 discussion group studies<br>- 2 randomized controlled trials	Not specified	Not specified	Comprehensions of drug label instructions	- Textual elements contributing to the correct interpretation of drug label instructions were:<br>o using explicit time periods in dosage instructions (mention the moment of intake specified by the hour of intake, dayparts, or mealtime anchors),<br>o using plain language. Medical jargon should be avoided (e.g., 'subcutaneously', 'inhalation') and complex words (e.g., 'prolonged or excessive exposure', 'tablets') should be substituted for simpler ones (e.g., 'under your skin', 'puff', limit your time in the sun', 'pills')<br>o presenting numbers in a numerical format<br>o providing drug label instructions in patients' native language.<br>- Multistep instructions per instruction line, using abbreviations and medical jargon seem to hinder the correct interpretation of drug label instructions.
**16.**	Marek et al., 2008 [16]	USA	To identify evidence-based interventions related to medication management and the community-dwelling older adult.	Book Chapter	Community care	Community-dwelling older adults	Not applicable	<u>Recommendations on medication procurement:</u><br>- Assess the patient's or caregiver's ability to procure medications: (1) Identify how and where the patient obtains and refills prescriptions; (2) assess how the patient pays for medications; (3) assess if medications doses are ever missed due to lack of funds.<br>- If the patient or caregiver has difficulty obtaining or refilling prescriptions, assist the patient with creating a system to procure medications via: (1) pharmacy delivery, (2) refill reminders or automatic refill service, (3) scheduling family or friends to pick up medications.<br>- If funds to purchase medication are a problem: (1) refer the patient to a social worker to obtain insurance coverage, or participation in drug company programs, (2) consult with the pharmacist regarding use of generic drugs, (3) consult the prescribing physician about availability of drug samples.<br><br><u>Recommendations on medication knowledge</u><br>- Assess the patient's or caregiver's knowledge of: (1)dose and frequency of medications taken; (2) special instructions related to medications, such as "take with food.", (3) if the patient uses an inhaler, understanding of the correct inhaler technique, (4) medication mode of action, (5) side effects to monitor and report.

								- Interventions related to medication knowledge include: (1) provide written instructions related to medications in large letters and bullet or list format, (2) tailor instructions to how the patient takes his or her medicine, (3) group information starting with generalized information, followed by how to take the medicine, and then the outcomes such as side effects to watch for and when to call the doctor, (4) use medication schedules or charts to reinforce instructions, (5) if the patient did not know important medication information at a previous encounter, review dose, time, side effects to monitor and report, and special instructions at the next visit.<br><br><u>Recommendations on physical ability</u><br>- Assess for decreased manual dexterity or vision impairment and its effect on the patient's ability to identify the correct medication, open medication containers, and prepare medications (e.g., breaking tablets) for administration:<br>- If the patient is unable to open or see the label and contents of each medication container, provide one of the following: (1) Pill box or other easy-open container. If the patient is unable to fill the pill box, identify someone who can assist him or her. (2) Medication calendar with pill box. (3) Blister packs. Consult the pharmacy about the availability of the drug in blister packs or non childproof containers. (4) If tablet breaking is required and the patient has difficulty doing it, consult with the pharmacist about tablets that are easier to break or tablets that are the correct dosage without requiring breaking.<br><br><u>Recommendations on cognitive capacity</u><br>- Teach the patient or caregiver the use of memory cues based on one of the following methods: (1) Clock time. Ask if the patient or caregiver is usually aware of the time of day or keeps track of time through a watch or clock. (2) Meal time. Ask if the patient eats meals at a regular time. (3) Daily ritual, such as using the bathroom in the morning, shaving, or hair combing.<br>- Provide memory-enhancing methods or devices such as medication calendar or chart, electronic reminder of alarm, voice-message reminder, telephone

								reminder, pill box, electronic medication dispensing service.<br>- Discuss dose simplification with the prescribing provider.<br><br><u>Recommendations on intentional non-adherence</u><br>- If the patient misses medication doses for reasons related to health beliefs, (1) explore with the patient his or her health concerns for not taking medication; (2) discuss the benefits of taking medication as prescribed; (3) provide positive reinforcement for taking medication as prescribed.<br>- For patients on high-risk medications, reinforce the danger of missing medication doses.<br>- If the patient misses medication doses for reasons related to medication side effects, (1) explore with the patient a plan to manage the side effects; (2) modify the regimen to reduce the side effects.
**17.**	Mullen et al., 2018 [17]	USA	To present evidence supporting best-practices for prescription drug labeling and educational materials.	Systematic review: 49 randomized controlled trials included	Not specified	Not specified	Comprehension, preferences and actual medication use	- Strong evidence for the use of plain, behavior-oriented language and explicit text.<br>- Other best-practices included the use of typographic cues (e.g. bolding, bullet points, headings, carriage returns, increased font size)<br>- Evidence was suggestive of establishing a standardized format and organization, such as the Universal Medication Schedule for pharmacy-generated container labeling and supplemental instructions.<br>- Research also clearly indicated the value of providing quantitative information in written prescription medication information to convey risks and benefits, instead of more qualitative, narrative statements.<br>- The use of pictograms and visual aids shows promise, particularly when standardized forms are used and paired with simplified text.
**18.**	National Institute for Health and Care Excellence, 2015 [18]	UK	This guideline covers safe and effective use of medicines in health and social care for people taking 1 or more medicines. It aims to ensure that medicines provide the greatest possible benefit to people by encouraging medicines reconciliation, medication review, and the use of patient decision aids.	Guideline	Health and social care	People taking 1 or more medicines	Not applicable	- Share relevant information about the person and their medicines when a person transfers from one care setting to another. This should include, but is not limited to, all of the following:<br>○ contact details of the person and their GP<br>○ details of other relevant contacts identified by the person and their family members or carers where appropriate – for example, their nominated community pharmacy<br>○ known drug allergies and reactions to medicines or their ingredients, and the type of reaction

								experienced (see NICE's guideline on drug allergy)<br>○ details of the medicines the person is currently taking (including prescribed, over-the-counter and complementary medicines) – name, strength, form, dose, timing, frequency and duration, how the medicines are taken and what they are being taken for<br>○ changes to medicines, including medicines started or stopped, or dosage changes, and reason for the change<br>○ date and time of the last dose, such as for weekly or monthly medicines, including injections<br>○ what information has been given to the person, and their family members or carers where appropriate<br>- Health and social care practitioners should discuss relevant information about medicines with the person, and their family members or carers where appropriate, at the time of transfer. They should give the person, and their family members or carers where appropriate, a complete and accurate list of their medicines in a format that is suitable for them. This should include all current medicines and any changes to medicines made during their stay.<br>- Offer all people the opportunity to be involved in making decisions about their medicines. Find out what level of involvement in decision-making the person would like and avoid making assumptions about this.<br>- Find out about a person's values and preferences by discussing what is important to them about managing their conditions and their medicines.<br>- Recognise that the person's values and preferences may be different from those of the health professional and avoid making assumptions about these.
**19.**	Nederlands Huisartsen Genootschap et al., 2020 [19]	The Netherlands	The purpose of the National Primary Care Collaboration Agreement is to optimise and secure the organisation of (pharmaceutical) care between pharmacists, GPs and home care nurses. This concerns care for the patient living at home with chronic	Report	Primary care	Community-dwelling patients taking chronic medication	Not applicable	- It is important that the patient knows what medication he is taking or is going to take, what the effect is, how to take it, what side effects he may suffer from after starting treatment.<br>- When discussing potentially reduced medication adherence with the patient, it is important to find out what the expectations, experiences, wishes, needs and bottlenecks are regarding medication use. Use motivational interview techniques.

			medication who receives home nursing care with support for medication use.					- Several structural measures are possible to improve adherence, including the use of a medication roll (possibly in combination with a medication dispenser), a weekly dosage box, a reminder app or a simplified medication schedule.
**20.**	Nederlandse Vereniging voor Klinische Geriatrie, 2020 [20]	The Netherlands	This guideline focuses on the best care for elderly people taking many different medications.	Guideline	Not specified	Older people with polypharmacy	Not applicable	To improve adherence, make use of:<br>- customised interventions tailored to individual patients;<br>- dosing once or twice daily with as few intake moments per day as possible;<br>- avoiding switching drug packs;<br>- combining comprehensible information and technical/practical interventions (such as medication on a roll) with behavioural interventions;<br>- medication assessment, asking about the patient's experiences and expectations with regard to the use of medication and discussing therapy adherence with the patient in a non-judgemental manner;<br>- good coordination between first and second line with preferably one point of contact in second line and one point of contact in first line;<br>- local and/or regional agreements on tasks and responsibilities, between first and second line and within first and second line according to the guideline 'Transfer of medication data';<br>- interventions tailored to specific target groups, such as patients discharged from hospital, nursing home or rehabilitation clinic, singles, depressed patients and patients with cognitive disorders.<br><br>Recommendations to prevent the likelihood of medication-related problems after discharge:<br>- Before discharge, provide written education to elderly patients and high-risk patients (who were followed intensively during admission). At a minimum, explain the indications for the medicines, the dosage schedule, method of use, changes made to (home) medication during admission and, if applicable, emphasise the importance of medication adherence. If necessary, involve the patient's informal carer.<br>- When educating the patient around discharge, apply teach back (ask the patient to repeat the information, to assess whether it has been properly understood).<br>- If a patient has been followed intensively during admission because they were classified as a high-risk

								patient on admission, transfer the information why the patient was classified as a high-risk patient to the next healthcare provider in the chain in accordance with the guideline Transfer of medication data in the chain.<br>- Before discharge, ensure that during medication verification, the medication list is reconciled with the patient. In addition, assess whether the discharge medication is appropriate (is the applicable protocols met, e.g. should adjuvant medication be stopped or started, etc.?). In doing so, also evaluate patient-related aspects such as fear of side effects, medication use problems and other utilisation problems.
**21.**	Nederlandse Vereniging voor Keel–Neus–Oorheelkunde en Heelkunde van het Hoofd–Halsgebied, 2017 [21]	The Netherlands	This guideline focuses on the best care for patients with dysphagia or underlying suffering at risk of dysphagia.	Guideline	Not specified	Patients with dsyphagia	Not applicable	Recommendations in case of swallowing problems:<br>- If possible, treat the underlying disease causing dysphagia medicinally.<br>- Assess whether medication is used that may affect dysphagia.<br>- If possible, change the medication to one that does not affect swallowing in consultation with the prescriber, pharmacist and patient.<br>- Evaluate whether orally prescribed medication can be taken safely.<br>- If necessary, adapt the form/route of administration of the medication to the dysphagia and state the reason for change on the prescription. There is a role for both the prescriber, pharmacist and patient here.
**22.**	Nunes et al., 2009 [22]	UK	This guideline covers medicines adherence in people aged 18 and over. It recommends how to encourage adherence to medicines by supporting and involving people in decisions about their prescribed medicines. It aims to ensure that a person's decision to use a medicine is an informed choice.	Guideline	Not specified	Adults receiving prescribed medicines and their families and carers	Medication adherence	Recommendations regarding involvement of patients in decisions about prescribed medicines and supporting adherence:<br>- Establish the most effective way of communicating with each patient and, if necessary, consider ways of making information accessible and understandable (for example, using pictures, symbols, large print, different languages, an interpreter or a patient advocate).<br>- Encourage patients to ask about their condition and treatment.<br>- Ask patients open-ended questions because these are more likely to uncover patients' concerns.<br>- Discuss with the patient why they might benefit from the treatment. Clearly explain the disease or condition and how the medicine will influence this.<br>- Explain the medical aims of the treatment to patients and openly discuss the pros and cons of proposed

								medicines. The discussion should be at the level preferred by the patient.<br>- Encourage and support patients, families and carers to keep an up-to-date list of all medicines the patient is taking. The list should include the names and dosages of prescription and non-prescription medicines and herbal and nutritional supplements. If the patient has any allergic or adverse reactions to medicines, these should be noted.<br>- Be aware that patients' concerns about medicines, and whether they believe they need them, affect how and whether they take their prescribed medicines.<br>- Ask patients what they know, believe and understand about medicines before prescribing new treatments and when reviewing medicines.<br>- Ask if the patient has any specific concerns about their medicines, whenever you prescribe, dispense or review medicines. These may include concerns about becoming dependent on medicines and concerns about adverse effects. Address these concerns.<br>- Discuss information on medicines with the patient rather than just presenting it. The discussion should take into account what the patient understands and believes about the condition and treatment.<br>- Patients differ in the type and amount of information they need and want. Therefore, the provision of information should be individualised and is likely to include, but not be limited to: what the medicine is, how the medicine is likely to affect their condition (that is, its benefits), likely or significant adverse effects and what to do if they think they are experiencing them, how to use the medicine, what to do if they miss a dose, whether further courses of the medicine will be needed after the first prescription, how to get further supplies of medicines.<br>- Be careful not to make assumptions about a patient's ability to understand the information provided. Check with the patient that they have understood the information. Information for patients should be clear and logical and, if possible, tailored to the needs of the individual patient.<br>- Suggest where patients might find reliable information and support after the consultation: for example, by providing written information or directing them to other resources

								- If a patient is not taking their medicines, discuss with them whether this is because of beliefs and concerns or problems about the medicines (intentional non-adherence) or because of practical problems (unintentional non-adherence).<br>- Because evidence supporting interventions to increase adherence is inconclusive, only use interventions to overcome practical problems associated with non-adherence if a specific need is identified. Target the intervention to the need. Interventions might include: suggesting that patients record their medicine-taking, simplifying the dosing regimen, using alternative packaging for the medicine, using a multi-compartment medicines system.<br>- Side effects can be a problem for some patients. If this is the case you should: discuss how the patient would like to deal with side effects, discuss the benefits, side effects and long-term effects with the patient to allow them to make an informed choice, consider adjusting the dosage, consider switching to another medicine with a different risk of side effects, consider what other strategies might be used (for example, timing of medicines).<br>- Ask patients if prescriptions charges are a problem for them. If they are, consider possible options to reduce costs.
**23.**	Palacio et al., 2016 [23]	USA	To evaluate the impact of Motivational Interviewing (MI) and of the MI delivery format, fidelity assessment, fidelity-based feedback, counselors' background and MI exposure time on adherence.	Systematic Review: 17 randomized controlled trials included	Not specified	Adults with chronic medications	Medication adherence	- Motivational Interviewing improves (self-reported and objective measures of) adherence to chronic medications after different lengths of exposure.<br>- Motivational Interviewing improved adherence when delivered by nurses or research assistants.
**24.**	Rijksinstituut voor ziekte- en invaliditeits-verzekering [24]	Belgium	Not applicable	Web Page	Not specified	Not specified	Not applicable	- The pharmacist is obliged to dispense the 'cheapest' medicine if the prescription is prescribed by substance name and dispensed in a community pharmacy.<br>- The "cheap prescribing" measure ensures that patients will have to pay less for their medicines.
**25.**	Rijksinstituut voor ziekte- en invaliditeits-verzekering [25]	Belgium	Not applicable	Web Page	Not specified	Not specified	Not applicable	- In case of a substance-name prescription, the doctor entrusts the care and responsibility to the pharmacist to carry out this prescription, taking into account the patient's needs in terms of continuity of treatment, price and availability.

**26.**	Rijksinstituut voor ziekte- en invaliditeits-verzekering [26]	Belgium	Not applicable	Web Page	Primary care	Not specified	Not applicable	- Every patient has the right to choose a family pharmacist.<br>- The 'family pharmacist' can keep the patient's medication schedule up-to-date and make it accessible to the other healthcare providers with whom he has a therapeutic relationship.<br>- The medication schedule should include all the drugs the patient is currently taking, both prescription and over-the-counter. The schedule should also include health products that could affect the effectiveness of the medicines (such as nutrients and foods) and medical devices.
**27.**	Ryan et al., 2014 [27]	Australia	To assess the effects of interventions which target healthcare consumers to promote safe and effective medicines use, by synthesising review-level evidence.	Overview of 75 systematic reviews	Consumers of medication irrespective of setting	Consumers, defined as any person using medicine(s), either a patient, carer or both	Medication adherence, medication knowledge, clinical and service-user outcomes, adverse events	- Medicines self-monitoring and self-management programmes appear generally effective to improve medicines use, adherence, adverse events and clinical outcomes; and to reduce mortality in people self-managing antithrombotic therapy. However, some participants were unable to complete these interventions, suggesting they may not be suitable for everyone.<br>- Other promising interventions<br>○ simplified dosing regimens: with positive effects on adherence;<br>○ interventions involving pharmacists in medicines management, such as medicines reviews (with positive effects on adherence and use, medicines problems and clinical outcomes) and pharmaceutical care services (consultation between pharmacist and patient to resolve medicines problems, develop a care plan and provide follow-up; with positive effects on adherence and knowledge).<br>- Several other strategies showed some positive effects, particularly relating to adherence, and other outcomes, but their effects were less consistent overall and so need further study.<br>○ practical strategies like reminders, cues and/or organisers, reminder packaging and material incentives: with positive, although somewhat mixed effects on adherence.<br>○ education delivered with self-management skills training, counselling, support, training or enhanced follow-up; information and counselling delivered together; or education/information as part of pharmacist-delivered packages of care: with positive effects on adherence, medicines

								use, clinical outcomes and knowledge, but with mixed effects in some studies;
**28.**	Scottish Intercollegiate Guidelines Network, 2012 [28]	Scotland	To present a template for a single discharge document that can be used as both the Immediate Discharge Document (in its core format) for every patient on the day of discharge, and as the final discharge summary/ letter (in its extended format) for more complex cases	Guideline	Transitional care	The discharge document is designed for patients who are discharged from hospital following an inpatient stay, i.e., a stay in hospital of one night or more. It could also form the basis of a discharge document for patients receiving treatment on a day-case basis.	Not applicable	The discharge document should include the following regarding medicines:<br>- Stopped medicines on discharge: name of medicine, formulation, strength of preparation, current dose, route of administration, frequency, reason for stopping.<br>○ Record all medicines that the patient was taking at the time of admission but was not taking at the time of discharge.<br>○ Describe the reason why each medicine listed here was stopped. This should include information on adverse reactions.<br>- New medicines on discharge: name of medicine, formulation, strength of preparation, current dose, route of administration, frequency, duration of treatment, number of days supply, reason for change to admission medicines, indications for new medicines.<br>○ Record new medicines prescribed during this hospital stay that are still being taken at the time of discharge. Do not include medicines that were only taken (i.e., that were started and stopped) during the patient's stay in hospital.<br>○ Record the number of days supply of each medicine that was given to the patient, carer or relative at the time of discharge. If none, record 'none'.<br>○ Provide a description of any aids to compliance (e.g., easy-open containers, medication charts, compliance devices, medication management service via carer) that have been provided to or are being used by the patient to assist with medicine intake.<br>○ If changes have been made to the formulation, strength, dose, frequency or route of administration of medicines that the patient was taking at the time of admission, record the reasons why these changes were made.<br>○ For medicines that are new, i.e., that were not being taken by the patient at the time of admission, describe what the new medicine has been prescribed for as this may not be clear to the GP or patient from the name of the medicine alone.

**29.**	Sharko et al., 2022 [29]	USA	To develop evidence-based recommendations for improving comprehension of quantitative medication instructions	Systematic review: 21 studies included	Not specified	Not specified	Comprehension-related outcomes (understanding, demonstrated accuracy in medication administration, recall of instructions, adherence, or preference for one or more information formats)	Recommendations for medication instructions:<br>(1) Format interventions<br>- Text instructions should use "time periods" to depict frequency instead of "times per day".<br>- Text instructions should use "mealtime anchors" to depict frequency instead of "times per day"<br>- Text instructions should use the "Take-Wait-Stop" format to communicate maximum daily dosing limits<br>(2) Visualization interventions<br>- Liquid medications instructions should use visualizations of liquid medication devices to depict specific doses<br>- Medication instructions should use visualizations tables with associated explanatory text<br>- Medication instructions should use visualization panels<br>- Medication instructions should use visualizations for populations with low health literacy<br>- Medication instructions should use visualizations for populations with limited English proficiency<br>- Medication instructions should use visualizations for non-English speaking populations<br>- Medication instructions should use images that have been validated<br>- Maximum dosing visualizations may be preferred to words/numbers alone
**30.**	Shrank et al., 2007 [30]	USA	To evaluate the evidence regarding the optimal content and format of prescription labels that might improve readability, understanding, and medication use.	Systematic review: 104 studies included	Not specified	Not specified	Readability, comprehension, and the capacity to take medications	- When optimizing content, patients prefer information about the indication for the medication, expected benefits, duration of therapy, and a thorough list of potential adverse effects, in addition to typical information identifying the drug's name, directions for use, and warnings.<br>- The evidence about label format supports the use of larger fonts, lists, headers, and white space, using simple language (avoid medication terminology) and logical organization (uniform medication scheme) to improve readability and comprehension.<br>- Evidence was not sufficient to support the use of pictographic icons.
**31.**	Simas da Rocha et al., 2021 [31]	Brazil	To evaluate the effectiveness of interventions designed to reduce problems with readability and comprehensibility in drug labels.	Systematic review: 17 studies included<br>- 14 experimental studies	Not specified	Medication users who are able to read	Readability and comprehensibility	- Increased font size yielded good results in the recognition and identification of medications mainly for the population with impaired vision, such as older adults.<br>- Users' comprehension improved when the layout of drug packages was modified by highlighting relevant

				- 3 quasi-experimental studies<br>- 1 observational study				information, such as medication name, dose, and instructions, making them clearer for medication users.
**32.**	Sletvold et al., 2020 [32]	Norway	To investigate the potential effect of pictograms on patient adherence to medication therapies.	Systematic review: 17 randomized controlled trials included	Primary and secondary healthcare settings	Patients on medication	Medication adherence	- Ten studies (58.8 %) reported a statistically significant effect, of the pictogram intervention in question, on patient adherence to medication therapies.<br>- Pictograms used in patient counselling support the proper use of medicines but should be presented in combination with written and/or oral information.
**33.**	Vilans [33]	The Netherlands	To provide information on tools to facilitate medication intake.	Website	Healthcare sector (general)	Older persons, chronically ill and disabled people	Not applicable	Aids to facilitate the intake of medicines:<br>- Pill or tablet splitter: Pill splitters and tablet splitters allow pills and tablets to be halved or further divided into the correct and measured size without any effort.<br>- Pill box: to keep track of pills and tablets to be taken in a given period<br>- Medication alarm: to remind a patient that it is time to take medication.<br>- Medicine dispenser with medication roll automatically offer a pouch of pills/ tablets in the right formulation and dosage at the right times. This prevents patients from taking a double dose, skipping a dose or taking medicines too early.<br>- Pill pusher: to push pills and tablets out of a blister pack.<br>- Tablet crusher<br>- Application for medication intake<br>- Eye-drop tools<br>- Ampoule opener
**34.**	Wali et al., 2015[34]	Canada	The primary aim was to systematically review the evidence on interventions for improving medication knowledge and adherence for low health literate populations.	Systematic review: 47 studies included<br>- 27 randomized controlled trials<br>- 8 non-randomized controlled trials<br>- 12 uncontrolled trials	Any healthcare setting	Low health literate populations	Medication knowledge and medication adherence	Interventions designed to support low health literate populations can improve patients' medication knowledge and adherence. The most effective interventions include<br>- additional sources of information, such as graphics, pictograms, icons, animations and verbal counselling in combination with written information<br>- individually tailored information<br>- the ability to easily navigate through information. Formats that improve navigation include bullets, subheadings, icons, bolding, underlining, larger font size and shorter words.

								- interventions that provided participants with information that they can have access to (e.g., pill cards, telephone reminder systems).
**35.**	Zomahoun et al., 2017 [35]	Canada	To assess whether motivational interviewing interventions are effective to enhance medication adherence in adults with chronic diseases and to explore the effect of individual MI intervention characteristics.	Systematic review: 19 randomized controlled trials included	Not specified	Adults with chronic diseases	Medication adherence	- Motivational Interviewing (MI) interventions might be effective at enhancing of medication adherence in adults treated for chronic diseases.<br>- Interventions that were based on MI only or those in which interventionists were coached during intervention implementation were the most effective.<br>- MI interventions that were delivered solely face to face were more effective than those that were delivered solely by phone
**36.**	Zorg voor Beter [36]	The Netherlands	To provide information on tools available for safe medication use and for administering medication independently.	Web Page	Hospital care and primary care	Patients with chronic conditions	Not applicable	Various tools are available for safe medication use and for administering medicines independently:<br>- Pill boxes<br>- Medication alarms<br>- Medication dispensers:<br>○ If the client has difficulty swallowing the tablet, a tablet pulveriser is a useful tool. The resulting powder can be mixed with food or drinks. Note: grinding medicines should only be done in consultation with the pharmacist or doctor.<br>○ With a tablet splitter, clients can split tablets in half themselves.<br>○ Medication roll: A dispenser in which the medicine roll is stores signals when it is time to take a pouch of medicines.<br>- Tools for opening medication packages: pill pusher, Open Aid Opener, ampoule opener or pill pen.

References

1. Bailey, S.C.; Annis, I.E.; Reuland, D.S.; Locklear, A.D.; Sleath, B.L.; Wolf, M.S. Development and evaluation of the Measure of Drug Self-Management. *Patient Prefer Adherence* **2015**, *9*, 1101-1108, doi:10.2147/PPA.S85411.
2. Belgische Vereniging voor Gerontologie en Geriatrie. *RICHTLIJN: HOE MEDICATIEBEGELEIDING (“MEDICATIE COUNSELLING”) BIJ OUDERE VOLWASSEN UIT TE VOEREN BIJ ZIEKENHUISONTSLAG*; 2020.
3. Brega AG; Barnard J; Mabachi NM; Weiss BD; DeWalt DA; Brach C; Cifuentes M; Albright K; West DR. *AHRQ Health Literacy Universal Precautions Toolkit, Second Edition.* ; Agency for Healthcare Research and Quality: 2015.
4. Capiau, A.; Foubert, K.; Van der Linden, L.; Walgraeve, K.; Hias, J.; Spinewine, A.; Sennesael, A.L.; Petrovic, M.; Somers, A. Medication Counselling in Older Patients Prior to Hospital Discharge: A Systematic Review. *Drugs & aging* **2020**, *37*, 635-655, doi:10.1007/s40266-020-00780-z.
5. Conn, V.S.; Ruppar, T.M.; Enriquez, M.; Cooper, P. Medication adherence interventions that target subjects with adherence problems: Systematic review and meta-analysis. *Research in social & administrative pharmacy : RSAP* **2016**, *12*, 218-246, doi:10.1016/j.sapharm.2015.06.001.
6. De Bodt, M.; Guns, C.; D'Hondt, M.; Vanderwegen, J.; Van Nuffelen, G. *Dysfagie. Handboek voor de klinische praktijk*; Garant: 2015.
7. Dietrich, F.M.; Hersberger, K.E.; Arnet, I. Benefits of medication charts provided at transitions of care: a narrative systematic review. *BMJ open* **2020**, *10*, e037668, doi:10.1136/bmjopen-2020-037668.
8. Farmaka. Delen of pletten van geneesmiddelen. Available online: https://farmaka.bcfi.be/nl/formularium/390#main (accessed on 4/03/2022).
9. Farmaka. Slikstoornissen bij ouderen: fysiologie, pathologie en aanpak. Available online: https://farmaka.bcfi.be/nl/formulariuminfo/brief/slikstoornissen-bij-ouderen-fysiologie-pathologie-en-aanpak (accessed on 14/03/2022).
10. Federaal agentschap voor geneesmiddelen en gezondheidsproducten (FAGG). Geneesmiddelen bewaren. Available online: https://www.fagg-afmps.be/nl/info_patienten/geneesmiddelen_bewaren (accessed on 15/02/2022).
11. Ha Dinh, T.T.; Bonner, A.; Clark, R.; Ramsbotham, J.; Hines, S. The effectiveness of the teach-back method on adherence and self-management in health education for people with chronic disease: a systematic review. *JBI database of systematic reviews and implementation reports* **2016**, *14*, 210-247, doi:10.11124/jbisrir-2016-2296.
12. Instituut Verantwoord Medicijngebruik. Tips voor hulpmiddelen en hulp beheer eigen medicatie. Available online: https://www.medicijngebruik.nl/zorginstellingen/werkmateriaal-overig/2118 (accessed on 15/02/2022).
13. Koninklijke Nederlandse Maatschappij ter bevordering der Pharmacie. Begrijpelijke medicijninfo voor iedereen. Available online: https://www.apotheek.nl/over-apotheeknl/begrijpelijke-medicijninfo-voor-iedereen (accessed on 14/03/2022).
14. Koninklijke Nederlandse Maatschappij ter bevordering der Pharmacie. Overdracht van Medicatiegegevens in de keten. Available online: https://www.knmp.nl/index.php/richtlijnen/overdracht-van-medicatiegegevens-de-keten (accessed on 16/07/2022).
15. Maghroudi, E.; van Hooijdonk, C.M.J.; van de Bruinhorst, H.; van Dijk, L.; Rademakers, J.; Borgsteede, S.D. The impact of textual elements on the comprehensibility of drug label instructions (DLIs): A systematic review. *PloS one* **2021**, *16*, e0250238, doi:10.1371/journal.pone.0250238.
16. Marek, K.D.; Antle, L. Advances in Patient Safety. Medication Management of the Community-Dwelling Older Adult. In *Patient Safety and Quality: An Evidence-Based Handbook for Nurses*, Hughes, R.G., Ed.; Agency for Healthcare Research and Quality (US): Rockville (MD), 2008.

17. Mullen, R.J.; Duhig, J.; Russell, A.; Scarazzini, L.; Lievano, F.; Wolf, M.S. Best-practices for the design and development of prescription medication information: A systematic review. *Patient education and counseling* **2018**, *101*, 1351-1367, doi:10.1016/j.pec.2018.03.012.
18. National Institute for Health and Care Excellence. Medicines optimisation: the safe and effective use of medicines to enable the best possible outcomes. Available online: https://www.nice.org.uk/guidance/ng5 (accessed on 13/07/2021).
19. Nederlands Huisartsen Genootschap (NHG); de Koninklijke Nederlandse Maatschappij ter bevordering der Pharmacie (KNMP); Verpleegkundigen en Verzorgenden Nederland (V&VN). *LESA Organisatie van zorg bij chronische medicatie. Handreikingen voor huisartsen, apothekers en wijkverpleging voor het maken van praktische afspraken over de organisatie van zorg rondom medicatiegebruik voor patiënten met wijkverpleging.*; Nederland, 2020.
20. Nederlandse Vereniging voor Klinische Geriatrie (NVKG). Polyfarmacie bij ouderen. Available online: https://richtlijnendatabase.nl/richtlijn/polyfarmacie_bij_ouderen/polyfarmacie_bij_ouderen_-_korte_beschrijving.html (accessed on 7/03/2022).
21. Nederlandse Vereniging voor Keel–Neus–Oorheelkunde en Heelkunde van het Hoofd–Halsgebied (NVKVO). Orofaryngeale dysfagie Available online: https://richtlijnendatabase.nl/richtlijn/orofaryngeale_dysfagie/medicatie_bij_dysfagie.html (accessed on 14/03/2022).
22. Nunes V; Neilson J; O'Flynn N; Calvert N; Kuntze S; Smithson H; Benson J; Blair J; Bowser A; Clyne W; et al. *Clinical Guidelines and Evidence Review for Medicines Adherence: involving patients in decisions about prescribed medicines and supporting adherence.* ; National Collaborating Centre for Primary Care and Royal College of General Practitioners.: London, 2009.
23. Palacio, A.; Garay, D.; Langer, B.; Taylor, J.; Wood, B.A.; Tamariz, L. Motivational Interviewing Improves Medication Adherence: a Systematic Review and Meta-analysis. *Journal of general internal medicine* **2016**, *31*, 929-940, doi:10.1007/s11606-016-3685-3.
24. Rijksinstituut voor ziekte- en invaliditeitsverzekering (RIZIV). 'Goedkoop voorschrijven'. Available online: https://www.riziv.fgov.be/nl/professionals/individuelezorgverleners/artsen/verzorging/Paginas/goedkoop-voorschrijven-20150101.aspx (accessed on 14/03/2022).
25. Rijksinstituut voor ziekte- en invaliditeitsverzekering (RIZIV). Voorschijven op stofnaam: regels voor de apotheker voor het uitvoeren van het voorschrift. Available online: https://www.riziv.fgov.be/nl/themas/kost-terugbetaling/door-ziekenfonds/geneesmiddel-gezondheidsproduct/afleveren/Paginas/voorschijven-stofnaam-regels-apotheker-uitvoeren-voorschrift.aspx (accessed on 14/07/2022).
26. Rijksinstituut voor ziekte- en invaliditeitsverzekering (RIZIV). Begeleiden van chronische patiënten als huisapotheker. Available online: https://www.riziv.fgov.be/nl/professionals/individuelezorgverleners/apothekers/Paginas/huisapotheker-begeleiden-chronische-patienten.aspx (accessed on
27. Ryan, R.; Santesso, N.; Lowe, D.; Hill, S.; Grimshaw, J.; Prictor, M.; Kaufman, C.; Cowie, G.; Taylor, M. Interventions to improve safe and effective medicines use by consumers: an overview of systematic reviews. *The Cochrane database of systematic reviews* **2014**, *2014*, Cd007768, doi:10.1002/14651858.CD007768.pub3.
28. Scottish Intercollegiate Guidelines Network (SIGN). The SIGN discharge document. **2012**.
29. Sharko, M.; Sharma, M.M.; Benda, N.C.; Chan, M.; Wilsterman, E.; Liu, L.G.; Demetres, M.; Delgado, D.; Ancker, J.S. Strategies to optimize comprehension of numerical medication instructions: A systematic review and concept map. *Patient education and counseling* **2022**, *105*, 1888-1903, doi:10.1016/j.pec.2022.01.018.
30. Shrank, W.; Avorn, J.; Rolon, C.; Shekelle, P. Effect of content and format of prescription drug labels on readability, understanding, and medication use: a systematic review. *The Annals of pharmacotherapy* **2007**, *41*, 783-801, doi:10.1345/aph.1H582.

31. Simas da Rocha, B.; Garcia Moraes, C.; Miyake Okumura, L.; da Cruz, F.; Sirtori, L.; da Silva Pons, E. Interventions to Reduce Problems Related to the Readability and Comprehensibility of Drug Packages and Labels: A Systematic Review. *Journal of patient safety* **2021**, *17*.
32. Sletvold, H.; Sagmo, L.A.B.; Torheim, E.A. Impact of pictograms on medication adherence: A systematic literature review. *Patient education and counseling* **2020**, *103*, 1095-1103, doi:10.1016/j.pec.2019.12.018.
33. Vilans. Hulpmiddelenwijzer. Available online: https://hulpmiddelenwijzer.nl/hulpmiddelen/bij/zorgen-en-verzorgen/medicijnen-nemen (accessed on 13/04/2022).
34. Wali, H.; Hudani, Z.; Wali, S.; Mercer, K.; Grindrod, K. A systematic review of interventions to improve medication information for low health literate populations. *Research in social & administrative pharmacy : RSAP* **2016**, *12*, 830-864, doi:10.1016/j.sapharm.2015.12.001.
35. Zomahoun, H.T.V.; Guénette, L.; Grégoire, J.-P.; Lauzier, S.; Lawani, A.M.; Ferdynus, C.; Huiart, L.; Moisan, J. Effectiveness of motivational interviewing interventions on medication adherence in adults with chronic diseases: a systematic review and meta-analysis. *International Journal of Epidemiology* **2017**, *46*, 589-602, doi:10.1093/ije/dyw273.
36. Zorg voor beter. Hulpmiddelen voor veilig medicijngebruik. Available online: https://www.zorgvoorbeter.nl/medicatieveiligheid/informatie-ouderen/hulpmiddelen (accessed on 15/02/2022).
