# Supplementary material for: The Development of Recommendations for Healthcare Providers to Support Patients Experiencing Medication Self-Management Problems
Source: Healthcare (Basel). 2023 May 25;11(11):1545. doi: 10.3390/healthcare11111545 (PMC10253050; doi:10.3390/healthcare11111545)
Supplement: Supplementary file 1 [file healthcare-11-01545-s001.zip › Supplementary_file_S4_English_translation_guide_recommendations.pdf]

RECOMMENDATIONS FOR HEALTHCARE PROVIDERS TO SUPPORT ADULT PATIENTS WITH PROBLEMS IN MEDICATION SELF-MANAGEMENT

University of Antwerp

NuPhaC
Nurse and Pharmaceutical Care

Contents

POSITIONING OF THE GUIDANCE DOCUMENT 3
INTRODUCTION/BACKGROUND 4
Medication self-management 4
Step 1: Picking up the medication from the pharmacy 4
Step 2: Knowledge and understanding of medication 5
Step 3: Organising and scheduling medication intake 5
Step 4: Medication intake 5
Step 5: Monitoring therapeutic and adverse effects of medication 5
Step 6: Maintaining proper and safe medication intake 5
Why is it important to address issues relating to medication self-management? 5
Why was the guidance document developed? 6
OBJECTIVE 7
END USERS 7
INTENDED PATIENT POPULATION 7
Why is it important to focus on patients with polypharmacy? 7
Why is it important to focus on hospitalised patients? 8
METHODOLOGY 9
STEP 1: Charting problems relating to medication self-management 9
STEP 2: Development of the guidance document 10
STEP 3: Validation of the guidance document 11
Selection and invitation of experts 11
Data collection and analysis 11
Patient involvement 13
PRACTICAL RECOMMENDATIONS 14
1. Picking up the prescribed medication at the pharmacy 14
1.1. The patient might be experiencing financial problems 14
1.2. The patient has an excessive, insufficient or no supply of medication 15
2. Knowledge and understanding of medication 16
2.1. The patient lacks knowledge of the medicines: name, indication, dosage, time of administration, method of administration 16
2.2. The patient is having trouble understanding information and/or instructions concerning the medication 18
3. Practical organisation and scheduling of medication intake 19
3.1. The patient is having trouble preparing the medication (or drug intake) 19
3.2. The patient is having trouble reading labels, package leaflets, information, instructions 20
3.3. The patient is having trouble establishing a daily routine 20
3.4. The patient is having trouble storing the medicines properly 21

4. Medication intake 21
4.1. The patient has swallowing problems 21
4.2. The patient is not using the medicines properly 22
4.3. The patient does not want to take the medicines 23
5. Supervision of desired and adverse effects of medication 23
5.1. The patient lacks knowledge about the desired and adverse effects (side effects and interactions) of the drugs 23
5.2. The patient is having trouble taking appropriate measures when side effects occur 24
6. Maintaining correct and safe medication intake 24
6.1. The patient has discontinued taking the medication prematurely 24
PROJECT GROUP 25
EXPERT PANEL 25
REFERENCES 28
APPENDICES 34
Appendix A: List of problems relating to medication self-management 34
Appendix B: Beliefs about medication questionnaire 36
Appendix C: Motivating conversational techniques 38

POSITIONING OF THE GUIDANCE DOCUMENT

These recommendations were developed to advise healthcare providers with regard to supporting hospitalised adult patients with polypharmacy in case of problems relating to the self-management of medication. The recommendations can also provide guidance when supporting other patient groups (e.g. non-hospitalised adults or patients taking fewer than five drugs). They were compiled based on scientific evidence, practical experience and/or expert consensus. The following aspects should be considered when implementing the recommendations:

- Supporting patients with regard to medication self-management requires an interdisciplinary approach, in which individual healthcare providers can contribute to improving patient medication self-management based on their own competences. In other words, the recommendations can be applied by a variety of healthcare providers (e.g. pharmacists, physicians, nurses) within their areas of expertise, preferably after coordinating with the members of each healthcare team.
- Supporting patients in medication self-management requires involving the patient (and/or informal carer) in the care process. The patient should be explicitly provided with the opportunity to inform the healthcare provider of any problems with self-management, as well as any personal experiences and preferences that affect drug use. To arrive at shared decision-making (i.e. decisions that the patient will support), the patient needs to have sufficient information about the issues at hand, the decisions to be made and the options available.
- Providing advice to patients regarding their medication (and/or its management) should not be limited exclusively to the time of hospital discharge. Patients should receive as much support as necessary in self-management during hospitalisation, as well as after discharge.
- It is not the intention to go through all of the recommendations in the guidance document systematically for every patient. The guidance document is intended as a resource for helping healthcare providers to intervene when there is evidence of problems in medication self-management. Patients admitted due to drug-related problems (e.g. medication errors, therapy non-adherence) should be regarded as having the highest priority.

INTRODUCTION/BACKGROUND

Medication self-management

Medication self-management is an important element of optimal medication management. It is regarded as an individual's ability to cope with the symptoms, treatment, physical and psychosocial consequences of a chronic condition, as well as with the associated lifestyle changes.[1] Self-management of medication consists of a series of tasks that individuals must perform successfully in order to manage their prescribed drugs themselves. Proper self-management requires knowledge, skills, motivation and behaviours to take drugs correctly and maintain their correct use over time for as long as indicated.[2]

The process of medication self-management has been translated into a model by Bailey et al.[2] As shown in Figure 1, the process begins with picking up the prescribed medication from the pharmacy. During the second step, the patient must understand and learn how to use the drugs safely and properly, and this requires knowledge concerning the medication (e.g. the name of the drug, its indication, the route of administration, and time of intake). The practical organisation and scheduling of drug intake is described in the third step of the model. The fourth step consists of the actual intake of the prescribed drugs. In the fifth step, patients monitor their drug intake and evaluate possible side effects, symptoms or usage problems related to their medicines, to take action if necessary. The sixth and final step involves maintaining a proper medication-intake routine in a safe and effective manner for as long as indicated, or deliberately discontinuing medication. This model implies that problems in medication self-management are directly related to a patient's therapy adherence.

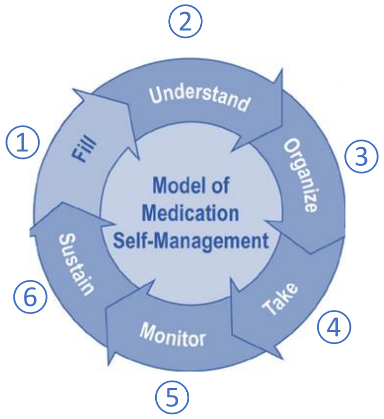

Figure 1: Model of medication self-management (Bailey et al., 2013)

Studies have shown that patients frequently experience problems during the various phases of the medication self-management process.

Step 1: Picking up the medication from the pharmacy

According to a systematic literature study, one in every six patients (17%) do not pick up all their prescribed medication at the pharmacy.[3] About 29% of all patients in the Netherlands who had been prescribed a drug by their GP did not pick it up from the pharmacy within seven days.[4] In a study of patients with polypharmacy (intake of ≥ 5 drugs) in Belgium, nearly half (48%) of the patients did not have all the necessary drugs on hand after discharge. Moreover, 31% did not have the necessary prescriptions available. A limited group of patients did not know whom they should contact to obtain a new prescription or did not have the resources (e.g. transport, assistance) needed to pick up drugs from the pharmacy.[5]

Step 2: Knowledge and understanding of medication

The ability to name and identify drugs, know their indications and understand how to take medicines from one's own drug regimen is necessary to the safe and effective use of medication, and it is therefore a fundamental part of medication self-management. However, patients may have trouble understanding drug instructions and information (labels, package leaflets).[6-9] Furthermore, it has been shown that patients with complex medication regimens have trouble naming their medication and its intended therapeutic goals.[10-12] In a survey of 95 home-care patients in the Netherlands, about 40% were not able to name their drugs, even with the help of a medication summary.[13] According to a study conducted in Flanders, 86% (n=334) of the patients with polypharmacy after hospital discharge experienced at least one knowledge deficiency (e.g. not knowing the name of a drug, its dosage or manner of administration).[5] In a Spanish study, participants had the lowest scores in the area of medication safety (e.g. precautions, warnings, side effects, contraindications, interactions).[12]

Step 3: Organising and scheduling medication intake

Patients have been shown to have trouble with organising the proper and timely intake of drugs. The integration of drug intake into the daily routine often goes wrong as well, and patients forget to take medicines at the proper time.[14] The dosing interval appears crucial in this regard: the fewer intake moments, the greater the likelihood of consistent and correct medication use.[15,16] Problems with packaging (e.g. opening child-resistant packaging and blister packs that are difficult to press or remove) have been documented in literature.[17-22] Patients also experience problems with storing their drugs correctly.[14,23,24]

Step 4: Medication intake

According to the World Health Organisation, 50% of all patients with chronic conditions do not take their drugs as prescribed. The more drugs patients have to take, the more complex will be the medication regimen they must manage and the more complex the administration can be, thereby increasing the risk of medication errors.[25] A systematic literature study by Mira et al. (2015) indicates that the prevalence of patients who manage their own drugs in the home setting and make at least one medication error varies from 12% to 59%, increasing to 75% for older people with complex therapeutic regimens.[26] Common problems include taking the wrong dosage, taking the wrong medicine, forgetfulness, and deliberately skipping drug intake.[5,26]

Step 5: Monitoring therapeutic and adverse effects of medication

When patients take their medication correctly, monitoring of therapeutic and adverse effects is necessary. This requires patients to have knowledge of the possible side effects, risks and warnings, and to act correctly in case of symptoms or signs related to drug intake. As evidenced by research, however, patients have limited knowledge of common side effects, precautions, contraindications and interactions.[7,12] When patients do experience side effects, they do not always take appropriate actions, instead discontinuing their medication intake (at least temporarily) or adjusting their drug regimens themselves.[5]

Step 6: Maintaining proper and safe medication intake

The final step for effective medication self-management is persevering in safe and appropriate medication usage throughout the entire duration of the prescription. Research has shown that, over time, patients reduce the use of their drugs or discontinue taking them prematurely.[27,28]

Why is it important to address issues relating to medication self-management?

Problems with medication self-management have an impact on **medication adherence**. Within this context, medication adherence is defined as the extent to which patients take their medicines as prescribed.[29] Medication non-adherence can be either intentional or unintentional. Intentional non-adherence implies that the patient consciously chooses not to follow the drug therapy.[30,31] For

example, patients might choose not to take their medicines because they do not consider them necessary or because they experience side effects. Unintentional therapy non-adherence indicates that patients want to follow the drug therapy but are confronted with practical difficulties.[30,31] For example, a lack of medication adherence may result from forgetting to take the drugs, not being able to open packaging or not being able to follow the instructions for taking the drug.[32] About 24% to 40% of all patients do not take their drugs as prescribed after hospital discharge.[5,33,34] Medication non-adherence can lead to poorer health results (e.g. increased mortality, reduced quality of life, loss of productivity), greater healthcare consumption, and higher healthcare expenditures.[35-40]

In addition to affecting medication adherence, problems with medication management have an effect on the **safe usage of medication**, and they can lead to medication errors that are hazardous to the patient. The prevalence of medication errors by patients in the home has been estimated between 12% and 59%. For older people with complex therapeutic regimens, this prevalence increases to 75%. Incorrect dosing, confusing drugs, not knowing the indications, taking drugs whose expiry date has passed and taking improperly stored drugs have been identified as common causes of medication errors,[26] which are accompanied by reduced drug effectiveness and additional expenses.[26]

As indicated by the above problems resulting from failure to manage medicines correctly, patients could benefit from support from healthcare providers with respect to medication self-management.

Why was the guidance document developed?

As highlighted in the preceding sections, patients can frequently experience problems during the various phases of the medication self-management process. It is therefore crucial to identify and address problems relating to medication self-management before hospital discharge, in order to prevent medication-related problems after discharge and thus promote medication adherence and safe medication usage at home.[41] Healthcare providers and patients have highlighted the importance of supporting measures to support the self-management of medication.[42] Training and supporting patients in the self-management of their medicines, as a partner in their care, is appropriate so that patients feel ready to be responsible for the management of their own medicines after discharge, thereby maximising therapeutic effects and minimising side effects.

However, in-hospital preparation and support for medication self-management and related problems have proven to be inadequate.[5] In most studies related to medication self-management, education on medication usage is delivered in a non-structured manner.[43-48] Support tools for healthcare providers to guide patients through problems with medication self-management are not available.

OBJECTIVE

This guidance document with recommendations aims to provide healthcare providers with a framework for supporting adult patients with polypharmacy in case of problems relating to medication self-management. These may include:

- Medication self-management problems that patients encounter in the home setting before hospital admission;
- Medication self-management problems that are detected by the patient and/or healthcare provider during hospitalisation;
- Medication self-management problems that patients expect to experience after discharge.

END USERS

The recommendations are intended for use by all healthcare providers involved in the medication management of adults (18+).

INTENDED PATIENT POPULATION

The recommendations apply to hospitalised adults (18+) with polypharmacy (5 or more drugs[49]) who self-manage all or part of their medicines after discharge.

- Managing medicines completely independently means that patients collect, store, prepare, set out and take their drugs themselves.
- Managing medicines partially independently means that certain tasks in the medication self-management process (e.g. picking up drugs, setting out drugs) are performed by a healthcare provider (either formal or informal), but that other tasks (e.g. taking drugs) are performed independently by the patient. If patients manage their medication together with informal healthcare providers or informal carers, these caregivers should be involved.

Why is it important to focus on patients with polypharmacy?

In Europe, the prevalence of polypharmacy amongst people 65 years of age and older has been estimated at 32.1%.[50] In 2015, the general prevalence of polypharmacy in Belgium was 20%. For women, prevalence rates varied from 11.2% in the age category 0–24 years to 49.5% for women 75 years of age and older. For men, these percentages ranged from 8.3% in the age category 0–24 years to 50.9% for men 75 years of age and older.[51] The prevalence of patients taking 10 or more drugs (excessive polypharmacy) amounted to 16.1%.[52]

Polypharmacy is often clinically indicated in specific conditions (e.g. diabetes mellitus, hypertension) and specific patient populations (e.g. patients with multimorbidity). 'Appropriate polypharmacy' recognises that patients may benefit from taking multiple drugs, provided their prescription is evidence-based and the patient's clinical condition, comorbidities and potential drug interactions are considered.[55] Nevertheless, patients with polypharmacy are at increased risk of problems with medication self-management, due to the greater number of drugs prescribed and more complex medication regimens that are more difficult to understand and manage.[56]

Why is it important to focus on hospitalised patients?

Studies have indicated that the transition from hospital to home is a critical period for patient safety, which often results in drug-related problems after discharge.[35,57] The prevalence of such drug-related problems after discharge varies from 14% to 49%,[58,59] and several factors are at play. For example, during hospitalisation, healthcare providers are responsible for the daily management and administration of drugs, while patients must manage their drugs themselves after discharge, often without adequate preparation.[35,60,61] Vulnerable patients (e.g. those prescribed a multitude of medications) are at increased risk of incorrect medication usage after discharge, especially if they do not receive support and guidance.[62] Several changes may have been made to the patient's medication regimen during hospitalisation.[63,64] Instructions describing changes in the medication regimen are not always explicitly and adequately communicated to patients.[60,62,65] After discharge, 54–82% of patients cannot name which medication was changed during admission, and 55% do not use the medication at home as initially intended at discharge.[66-68]

Note: *The guidance document was developed with a focus on hospitalised adults with polypharmacy. The recommendations can nevertheless also provide guidance for other patient groups (e.g. non-hospitalised adults or patients taking fewer than five drugs).*

METHODOLOGY

As shown in Figure 2, the development and evaluation of the recommendations for healthcare providers to support medication self-management problems proceeded through several stages.

Figure 2: Stepwise methodology plan

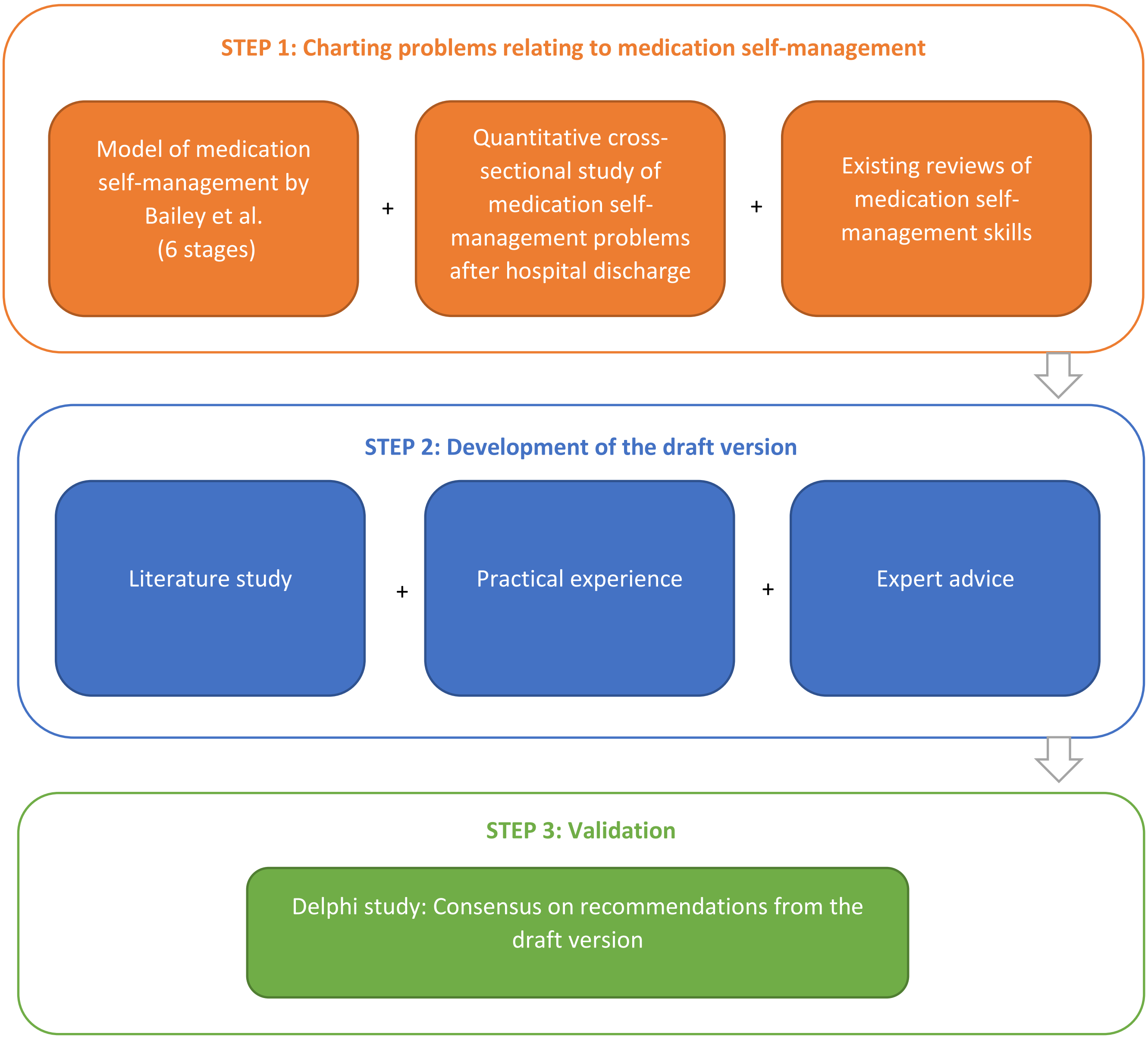

STEP 1: Charting problems relating to medication self-management

A list of problems relating to medication self-management was compiled, based on literature (Appendix A). For this purpose, we proceeded from the model of medication self-management developed by Bailey et al., which divides the medication self-management process and related problems into six stages.[2] The contents of this model were then compared and complemented with the results from a recently conducted quantitative cross-sectional study of medication management problems in patients with polypharmacy after hospital discharge.[5] Finally, validated tools for assessing medication management skills were consulted.[69-72] Problems with the skills needed in this regard can be understood as medication self-management problems.

STEP 2: Development of the guidance document

A draft version containing recommendations was elaborated, based on literature study and practical experience. In an early stage, guidelines on medication management were searched in the following databases: National Institute for Health and Care Excellence (NICE) guidelines, Guideline International Network, SIGN guidelines, NHG-richtlijnen, Richtlijnendatabase (NL), Clinical Practice guidelines (CPG), Ebpracticenet and the Federaal Kenniscentrum voor de gezondheidszorg (KCE). Combinations of the following search terms were used: 'medicines', 'medication', 'medicatie', 'geneesmiddelen', 'geneesmiddelentherapie', 'medication management', 'medicines management', 'medicatie management', 'self-management', 'zelfmanagement', 'polypharmacy', 'polymedication', 'polyfarmacie', 'compliance', 'adherence', 'therapietrouw'.

In a second stage, PubMed was searched for existing literature overviews and guidelines focussing on medication self-management and related problems. Various combinations of search terms were used to this end:

Concept	Search terms
Polypharmacy/medication	'polypharmacy[MeSH]','polypharmacy', 'polymedication', 'multiple drug therapy', 'multidrug therapy', 'multidrug regimen', 'multiple medication', 'multiple drug*', 'medication', 'medicine*', 'drug*'
Self-management	'self manag*', 'self-management', 'self-management[Mesh]', 'self-management support'
Picking up prescribed medication at the pharmacy	'obtain', 'purchase', 'access*', 'prescription'
Knowledge and understanding of the medication	'medication knowledge', 'drug knowledge', Patient Medication Knowledge[Mesh], 'Patient drug knowledge', 'knowledge', 'comprehension', comprehension[Mesh], 'dosage', 'dose', 'administration route', 'indication', 'readability', 'comprehension', 'labels', 'information', 'instruction'
Medication intake	'dysphagia', 'presbyphagia', 'swallowing problems', 'swallowing difficulties', 'swallowing disorder', deglutition disorders[MeSH]
Supervision of therapeutic and adverse effects	'adverse effect*', Drug-related side effects and adverse effects[MeSH], 'therapeutic effect', 'precaution', 'side effect', 'contraindication*', 'contra-indication*', contraindications[MesH], 'interaction', Drug Interactions[MeSH], 'warning*', 'monitoring'
Maintenance of correct medication intake	'persistence', 'medication persistence', 'medication adherence[MeSH]', 'medication adherence', 'drug adherence', 'medication compliance', 'Medication Nonadherence', 'Medication Non-Adherence', 'Medication Non Adherence', 'Medication Noncompliance', 'Medication Non-Compliance', 'Medication Non Compliance'
Population	'patient*', 'client*', 'consumer*', 'adult', adult[MeSH]
Filters	Article Types: Systematic review, Review, Guideline<br>Language: English, French, Dutch

The search results were screened according to title and abstract. Guidelines and overviews published in English, French or Dutch were eligible for inclusion. To be included, the abstract had to clearly report on recommendations and/or interventions to improve medication management for adult patients in hospital or primary care. The full text of the articles was then read through. The effectiveness of interventions aimed at supporting self-management was seldomly evaluated. Most of the few existing studies are not well-designed clinical trials. This guidance document therefore does not include any assessment of the effectiveness or the reliability of the recommendations. It provides an overview of the interventions described, including the source references.

In the third stage, the information retrieved was supplemented with information from grey literature. To this end, information was sought on the websites of hospitals and healthcare facilities, as well as on websites intended for healthcare providers (e.g. FAGG, Instituut voor Verantwoord Medicijngebruik [Institute for the Responsible Use of Medicine], Zorg voor Beter [Caring for Better]).

STEP 3: Validation of the guidance document

A Delphi study was conducted to achieve consensus on the recommendations formulated to help healthcare providers support patients in case of problems relating to medication self-management. Experts were asked to assess the relevance and clarity of the recommendations on a scale from 1 (extremely irrelevant/unclear) to 4 (extremely relevant/clear). The opportunity was provided to propose changes to the original recommendations or to add new ones based on practical experience and expertise. An assessment form was developed for assessing the guidance document with recommendations. This form was reviewed and adjusted by the project group.

Selection and invitation of experts

Experts were selected through a targeted sample. The participants had to meet the following selection criteria:

1. Belonging to one of the following professional groups: nurses, doctors or pharmacists
   AND
2. Working in Belgium or the Netherlands
   AND
3. Having an established national or international profile in the field of medication management/patients with polypharmacy, recognised by scientific publications, policy reports and/or extensive participation in specialised thematic conferences, meetings or interest groups
   OR
   Being involved in the medication management of patients with polypharmacy in daily practice, as stakeholders who would use the guidance document in daily practice
   AND
4. Having good Dutch language skills to complete the surveys

The size of the group of experts required to participate in the Delphi survey did not depend on statistical power, as representativeness in such surveys is assessed according to the quality of the sample, rather than its size.[73] Each member of the project group made suggestions for experts who were included in the expert panel. Feedback and agreement on the list of experts was obtained amongst project members. In choosing the experts, a balanced representation of all professional groups, settings (academic/clinical practice) and countries (Belgium/Netherlands) was sought. Project group members were also given the opportunity to participate as experts in the Delphi study.

Experts were invited by email to participate in the study. The invitation was accompanied by the necessary information on the design, objective and conduct of the study. If no response was obtained after two weeks, a reminder email was sent. Afterwards, each expert who agreed to participate received an invitation for a feedback round with a response deadline of four weeks from the day of the invitation. Each round was followed by a period for summarising and analysing the responses and integrating the results into the next Delphi round. Afterwards, another invitation for a subsequent feedback round was sent to all experts from the previous round.

Data collection and analysis

Experts were initially asked to provide information on their demographics, professional background and specific areas of expertise for descriptive purposes and to confirm their eligibility in the first round of surveys.

The first round of questions consisted of:

- Assigning a score for the relevance and clarity of each item in the guidance document;
- Assigning a score for the usability and feasibility of the current design of the guidance document;
- Formulating comments or suggestions (optional);
- Formulating new recommendations (optional).

Results from this round were reported to participants in the next round through descriptive percentages and frequency distributions. The retention or deletion of items in the next round was guided by a few decision rules. Consensus for relevance was defined as ≥ 80% of the experts in the sample having assigned a score of 3 or higher. The item was then retained. If ≥ 80% of respondents assigned a score of 2 or lower for a recommendation, the recommendation was deleted from the item list.

If ≥ 80% of respondents assigned a score of 2 or lower for clarity of wording, the wording of the respective recommendations included in the list was refined. The comments from the experts were then used to revise the formulation of each corresponding recommendation. The reformulated items were resubmitted to the experts in the next round of questions.

If ≥ 80% of respondents assigned a score of 3 or higher for clarity of wording, the item was accepted. Based on the comments/suggestions from the experts, changes could still be made to enhance the clarity of the wording for items that had been accepted.

- If the comments/suggestions led to non-fundamental changes (e.g. changes in sentence structure, addition of punctuation marks), the item was accepted with changes and not resubmitted in the next round of questions. Experts did have the opportunity to make additional comments on these changes if they wished.
- If comments/suggestions led to fundamental changes in the item despite its having been accepted, the item was resubmitted to the experts in the next round of questions.

The second round of questions consisted of a revised item list, including the scores of the initial items and new items from the first round. Experts were invited to:

- Revise their opinions by assigning new scores for relevance and clarity and adding comments for items that did not reach consensus on relevance in the first round;
- Assign a score for the relevance and clarity of each *new* item;
- Formulate comments or suggestions (optional).

The third round consisted of presenting consolidated feedback for all items on which consensus on relevance was not reached in previous rounds, with the experts having the chance to revise their opinions accordingly with:

- New relevance and clarity scores for the remaining items;
- The formulation of comments and suggestions (optional).

The Delphi rounds were discontinued once consensus was reached for more than 80% of the recommendations with regard to relevance. This rule was applied starting with the first round if no new recommendations were proposed or with the second round after the experts had assigned scores for the proposed new recommendations. A maximum of three rounds of questions were organised. If no consensus was achieved on certain recommendations after three rounds, these recommendations were no longer included in the guidance document.

Patient involvement

Simultaneous with the second Delphi round, the guidance document with recommendations was submitted to a diverse panel of patients with polypharmacy (e.g. differing age, differing educational level, differing types of chronic conditions). The patient panel was asked to formulate one-off feedback on the guidance document. First, a questionnaire was used to query some socio-demographic information (sex, age, education, number of drugs taken, medication management in the home setting). This information was used to describe the characteristics of the sample and to confirm the suitability of patients for participation in the study. In addition, statements were used to gauge what patients generally thought of the guidance document (and the recommendations contained within it). Patients were asked to indicate in the guidance document the recommendations they considered useful (green highlighting) and which they did not consider useful (red highlighting). If patients had no opinion on a particular recommendation, they were instructed to leave the recommendation blank (no highlighting). Open-ended questions were used to identify which recommendations from the guidance document patients might not have considered useful, along with their reasoning. Subsequent questions then used to probe for recommendations regarding the management of medication in the home setting that patients felt were currently missing from the guidance document. Moreover, space was provided for additional comments from patients on the guidance document. The final group of questions probed the extent to which patients found it difficult to understand the recommendations.

The comments (e.g. reasons for deeming certain recommendations not useful) and suggestions (e.g. new recommendations) from patients were summarised and incorporated into a narrative report and fed back to the expert panel of healthcare providers during the third Delphi round. If ≤ 70% of the patients considered a recommendation useful (or not useful), the expert panel of healthcare providers was asked to reassess the relevance of the recommendation during the third Delphi round.

PRACTICAL RECOMMENDATIONS

Note: Recommendations based on practical clinical experience are indicated with the letters **ExpB** (experience-based).

1. Picking up the prescribed medication at the pharmacy

1.1. The patient might be experiencing financial problems

1.1.1. If there is a suspicion or indication of financial problems, or if there are opportunities to reduce the cost of drugs, ask about the extent to which patients are interested in recommendations on the financial aspect of their drugs and how to address any financial difficulties in purchasing drugs (**ExpB**).

- If the patient is not interested in recommendations on the financial aspect of medication, no further action should be taken.
- If the patient is interested, see the following recommendations.

1.1.2. **Belgium:** Inform the patient that prescription drugs are subject to co-payment (i.e. a patient pays a personal share). Refer the patient to the hospital's social services department, family pharmacist or mutual insurance company for guidance and advice on reimbursement schemes, increased allowances or supplementary insurance options under which the co-payments required of the patient are reduced (**ExpB**). Information on the reimbursement of drugs is available on the website of the National Institute for Health and Disability Insurance (RIZIV) (in Dutch or French only), or on the website of the Belgian Centre for Pharmacotherapeutic Information (BCFI) (in Dutch of French only).

Netherlands: Inform the patient that the vast majority of prescription drugs are reimbursed under the basic health insurance. The preference policy of health insurers means that, in general, no additional payment is required of patients. In this preference policy, a health insurer designates a preferred drug within a group of similar drugs (i.e. drugs with the same active ingredient) to be reimbursed. In the Netherlands, the patient is subject to a required deductible. More specifically, patients are required to pay at least the first €385 (2022) of drug and/or hospital costs themselves. Only after this deductible has been paid are the costs covered by the basic health insurance. If necessary, refer the patient to the health insurer for counselling and advice on reimbursement schemes, increased allowances or supplementary insurance options (**ExpB**). Information on drug reimbursements is available at:

- The website of the national government
- The website 'medicijnkosten.nl' (in Dutch only). On this website, one can check whether a drug is included in the basic health insurance package; which costs count towards the patient's deductible; what the patient's co-payment will be (if any); and whether there is a less expensive medicine for which the patient will be reimbursed in full.

1.1.3. **Belgium:** Determine whether the patient is taking the least expensive variant of drugs from the point of view of the patient's personal contribution.[74]

- The RIZIV's online search engine 'Least expensive drugs' (in Dutch or French only) can be used to identify the specialities belonging to the group of 'least expensive' drugs for each substance name.[75,76]
- In the application or on the website of the Belgian Centre for Pharmacotherapeutic Information (BCFI; in Dutch or French only), the 'least expensive drugs' in the directory are indicated by the symbol .[76]

1.2. The patient has an excessive, insufficient or no supply of medication

Patients may not have the required medication at home due to lack of repeat prescriptions, or they may be having trouble in picking up drugs from the pharmacy.[5] It is also possible for patients to have double medication at home after hospitalisation (i.e. they have medication from before hospitalisation and medication given by the hospital). Patients unnecessarily use both drugs at the same time, as they do not know they are equivalent drugs, due to differences in the name or appearance of the drugs.

1.2.1. **Belgium:** Upon discharge, provide a supply of medication for up to three days to bridge the period between hospital discharge and a visit to the community pharmacy.[78,79]

- Inform the patient about the number of days for which the medication was supplied.[78,79]
- Advise the patient not to take medication left at home during this period to avoid double intake (**ExpB**).
- In some cases, a different brand of medicine is used in hospital than at home. If applicable, indicate the home medication to which the hospital's formulary product corresponds.[78]
- Inform the patient about which drugs were already administered on the day of discharge to avoid double intake.[78]
- Ensure that necessary prescriptions were sent electronically upon discharge, especially in the case of newly started drugs (**ExpB**).

> *Note:*
> - *If an outpatient pharmacy is present in the hospital, patients can immediately pick up their discharge medication there and, in principle, no medication supply needs to be given.*
> - *This recommendation does not apply in case of continued use of home medication during hospitalisation or in case of delivery of home medication during admission at the start of new medicines.*

Netherlands: Advise the patient to pick up discharge medication at the hospital's outpatient pharmacy or their own community pharmacy.

- Inform the patient about which drugs were already administered on the day of discharge to avoid double intake.[78]
- Ensure that necessary prescriptions were sent electronically upon discharge, especially in the case of newly started drugs (**ExpB**).

> *Note: This recommendation does not apply in case of continued use of home medication during hospitalisation and in case of delivery of home medication during admission at the start of new medicines.*

1.2.2. Advise patients to keep track of when repeat medication or repeat prescriptions are needed by using a calendar, a diary or an app with reminders.[80] Advise the patient to

contact the physician by telephone or schedule a consultation in a timely manner in the absence of the necessary prescriptions. The physician can then send an electronic prescription in the patient's name that the patient can pick up at the pharmacy (**ExpB**).

Community pharmacies in the Netherlands provide an automatic repeat service, whereby the pharmacy periodically dispenses repeat medication in case of chronic drugs. Upon discharge, the hospital should inform the community pharmacy about medication so that repeat service can be coordinated accordingly (**ExpB**).

1.2.3. Estimate the patient's ability to obtain drugs by asking how and where the patient obtains new prescriptions and/or drugs.[81] If the patient is having trouble in obtaining or picking up drugs, options such as arranging home delivery of drugs by the pharmacy or organising assistance in picking up drugs by an informal carer or family help should be considered in consultation with the patient and arranged, if desired.[81]

2. Knowledge and understanding of medication

2.1. The patient lacks knowledge of the medicines: name, indication, dosage, time of administration, method of administration

2.1.1. During hospitalisation, evaluate what patients already know regarding their medication.[74,78]

- Ask the patient to name the following elements for each medicine: name of the medicine, why the medicine should be taken (indication), method of administration, quantity, dosage and dosing interval.
- Most importantly, the patient should know the drug's indication. The other elements should be stated on the medication schedule (see Section 2.1.2), which the patient should understand.
- If a patient does not have sufficient knowledge about a particular drug, specific information about that drug can be provided. For elderly people with cognitive problems, it is advisable to involve an informal carer in providing drug-related information.

2.1.2. Provide the patient with an up-to-date medication schedule upon discharge, and include the following information for all current medicines.[78,79,82,83]

- Name, including dosage, dosage unit and formulation
- Indication
- Dosage frequency (per day, per week, per month)
- Time of administration (+ state whether it should be taken with food or on an empty stomach)
- Amount + unit per administration
- Route of administration
- State the ending date for medicines with a fixed duration (e.g. pain medication)

Use the medication schedule as a guide when informing patients about their drugs. This schedule should be clear and include only terms that the patient understands. Explain to the patient how to apply the schedule.[78,83]

Explain all medication changes that occurred during the hospital stay (what was stopped, started or changed) and the underlying reasoning.[78,79,82]

- In consultation with the physician, draft a pharmaceutical discharge letter and/or, if possible, use colours to provide a visual indication of what is new and what has changed on the medication schedule.[78] A 'new' drug is hereby defined as any drug that the patient was not using at the time of admission or any drug that the patient was using at the time of admission but whose formulation, dosage, frequency or route of administration has changed.[79]
- Note all discontinued drugs:[78,79,82] do not summarise them in the actual medication schedule, but separately in order to avoid confusion.

Discuss the items below for all newly started drugs and for all drugs for which additional information is indicated (e.g. high-risk medication, drugs that were involved in the reason for admission):[78]

- Provide information on the indication (why a drug should be taken).[78]
- Provide information on dosage and dosing interval (per day, per week), along with quantity and unit per administration, time of administration, and route of administration.[78]

> *Note: The guideline entitled* Overdracht van medicatiegegevens in de keten [Transfer of medication data in the chain; in Dutch] *define the medication data that must be included in the schedule, including medication that has been changed or discontinued.*[84]

2.1.3. Verify whether the patient has understood the medication schedule and/or drug-related information, and whether the patient considers them clear and complete.[86,90]

- Use the 'teach-back' method:[74,78,85-87] ask patients to repeat in their own words what has been explained to them about their medication (and medication schedule).
- If necessary, ask the patient to answer a specific question using the medication schedule (e.g. 'When should you take Drug X?'). This will make it possible to detect any problems related to reading and interpreting the schedule.
- Determine whether any information is missing according to the patient.
- Determine whether the patient has additional questions and/or whether there is anything that needs to be repeated or explained again.

2.1.4. Efforts should be made to implement an operational electronic health platform on which at least an up-to-date medication schedule can be accessed by all healthcare providers, both inside and outside the hospital. In the meantime, it is advised to send a medication schedule home with the patient/informal carer upon discharge. Explain that this medication schedule should be shown to all healthcare providers who need to be informed about it (e.g. GP, family pharmacist, home nurse), and encourage the patient/informal carer to have an up-to-date medication schedule with them whenever contacting a healthcare provider. If a healthcare provider makes any changes to the medication schedule, the healthcare provider should provide the patient with an adjusted, updated schedule (**ExpB**).

2.1.5. Inform the patient of the right to select a 'family pharmacist'. This means that the patient can designate a community pharmacist as a reference pharmacy. The main task of a family pharmacist is to guide and continuously monitor the patient's medicinal treatment. Other tasks of the family pharmacist can include keeping the medication schedule up to date and

making it accessible to other healthcare providers with whom the patient has a therapeutic relationship. Advise the patient to discuss this with the community pharmacist.[88]

2.2. The patient is having trouble understanding information and/or instructions concerning the medication

2.2.1. Determine the most effective way to communicate with each patient and, if necessary, consider ways to make information more accessible and understandable.[74,85]

- Adapt the mode of communication to the needs of the patient: ask what the patient needs.
- Determine whether the patient can read before providing drug instructions in writing.
- If possible, combine written and oral drug instructions with visual elements (e.g. pictograms or images,[89-92] or audio or video materials.[74,85,92] Pictograms can be printed from 'bijsluiterinbeeld.nl'. The KIJKsluiter Foundation provides information through animated videos and icons (https://www.kijksluiter.nl/).
- If possible, provide instructions or instructional videos in the patient's native language, and use an interpreter if possible.[74,85] Apotheek.nl provides instructional videos on the proper use of drugs in Dutch, English, Turkish, and Arabic (https://www.apotheek.nl/videos).
- For elderly people with cognitive problems, an informal carer should be involved in providing drug-related information.

2.2.2. Drug instructions should be brief, clear and specific. Avoid medical jargon (e.g. 'subcutaneous', 'inhalation') and complex words (e.g. 'prolonged exposure to the sun', 'tablets'), replacing them instead with simpler words (e.g. 'under the skin', 'inhalation', 'short time in the sun', 'pills').[93,94]

2.2.3. For written drug instructions, use large print, bullet points or a list format.[81]

2.2.4. Link medication use to specific time periods during the day to clarify the dosing interval.[89,91,93,94] The indication of time periods (morning, afternoon, evening, bedtime) is usually contained in the medication schedule. If necessary, the time periods can be specified in greater detail for the patient (e.g. on waking, before breakfast, before lunch, with lunch, before dinner, before bed).

2.2.5. Do not make any assumptions about a patient's ability to understand the information provided, but ask for feedback. Verify whether the patient has understood the drug-related information and instructions, and whether the patient considers them clear and complete.[86,90]

- Use the 'teach-back' method.[74,78,85-87]
  - Ask patients to repeat in their own words what has been explained to them about their drugs.
  - Ask patients to demonstrate any new self-care tasks that they will have to perform at home (e.g. using an inhaler, administering a subcutaneous injection, preparing medicines according to the medicine schedule).
- Determine whether any information is missing according to the patient.
- Determine whether the patient has additional questions and/or whether there is anything that needs to be repeated or explained again.
- Encourage patients to ask questions by asking: 'What questions do you have?'

- Ask whether the patient would like to receive additional written information (e.g. brochures, if available).

2.2.6. Indicate where patients can find reliable information and support after hospitalisation. For example, send written information home with them or refer them to other sources of information (e.g. GP, pharmacist, drug package leaflets, websites such as apotheek.nl).[74,78]

3. Practical organisation and scheduling of medication intake

3.1. The patient is having trouble preparing the medication (or drug intake)

Preparation includes such tasks as opening drug containers, breaking or splitting drugs in half, filling a medication box and using tools to administer eye drops.

3.1.1. In consultation with the patient, discuss whether assistance from an informal carer or home-care nurse is needed to prepare and set out drugs for a certain period of time. Arrange such help for the patient, if necessary (**ExpB**).

Recommendations 3.1.2 to 3.1.6 are applicable only if the preparation, setting out and taking of drugs are performed by the patient.

3.1.2. Ask patients whether they are having trouble opening drug packaging. In case of problems with opening the packaging, ask the pharmacist whether the medication is also available in another form of packaging that is easier to open.[80]

3.1.3. Advise the patient to use tools to open various types of containers (e.g. pill pusher, ampoule opener, pill pen). These tools are usually available from the pharmacist (including the family pharmacist).[80,95,96]

> *Note: Pill pushers should not be used for all medication. The pharmacist (or family pharmacist) can provide additional information about the application of such tools.*

3.1.4. Consult with the pharmacist about tablets that are easier to break (if breaking tablets is allowed) or tablets with the correct dosage so that breaking is not necessary.[81]

3.1.5. Advise the patient to use a tablet or pill splitter to halve or further divide the drugs into the appropriate and measured size (only if drug division is allowed). This tool is usually available from the pharmacist.[80,95,96]

3.1.6. Advise the patient to use eye-drop tools if the patient has trouble:
- Aiming/positioning the vial;
- Grasping the vial (not enough grip);
- Holding the hand with the drops still;
- Squeezing the vial (not enough strength);
- Keeping the eye open and not blinking;
- Pressing the tear duct shut after administering the eye drops.

Tools are usually available from the pharmacist.[80]

3.2. The patient is having trouble reading labels, package leaflets, information, instructions

3.2.1. Determine whether the patient uses/needs glasses (**ExpB**).

3.2.2. Refer the patient to instructional videos on drugs if reading drug-related information is difficult. Apotheek.nl provides short videos (in Dutch) with explanations by pharmacists for many drugs (https://www.apotheek.nl/videos/medicijnen?). KIJKsluiter provides animated videos in which the most important information from the package leaflet is explained in comprehensible language (https://www.kijksluiter.nl). (**ExpB**)

3.2.3. For documents to be drafted or document types that may be changed, the following principles can be used to improve legibility:

- Use sans serif fonts (e.g. Century Schoolbook or Helvetica).[91]
- Use a minimum font size of 12.[91,92,97]
- Structure instructions or information:[91,92,98]
    - Use boldface to emphasise important information.
    - Use summary lists instead of full paragraphs of text.
    - Use headings.
    - Provide adequate white space.
    - Use visual elements (e.g. pictograms and symbols). A package leaflet containing pictograms for each drug can be created through bijsluiterinbeeld.nl.

3.2.4. In case of illegible information on packaging: if possible, affix a sticker to each drug package stating the most important usage information (name, dosage, instructions for use) in a more readable font and size (**ExpB**).

3.3. The patient is having trouble establishing a daily routine

3.3.1. Discuss with the prescriber(s)/pharmacist whether the medication regimen can be simplified,[86,99] while trying to limit the number of intake times per day.[86]

3.3.2. Advise the patient to link taking medication, if possible, to fixed, recurring times in the day (e.g. before a meal, before brushing teeth or at bedtime).[100]

3.3.3. Inform patients about the possibility of setting medication out in places that remind them of intake, thereby linking intake to a habit or fixed routine of the patient. For example, medication on the bedside table is medication at bedtime; medication on the breakfast table is medication to be taken in the morning, and so forth. (**ExpB**)

3.3.4. In consultation with the patient, review the use of tools that can help with taking drugs in a timely manner/establishing a daily routine. However, evaluate the suitability of the tools for each patient individually.

- Pill box for setting drugs out by day and by time of intake (morning, afternoon, evening): various shapes and formats are possible, with and without an alarm function.[95,96]
- Pill timers, watches or alarms: a signal reminds the patient that it is time to take the medicines. [95,96]

- Digital applications for medicine usage: patients can maintain an overview of when which drugs should be taken. Most applications give a reminder at the time when the drugs should be taken.[96]
- Medication role: for 'drugs on a roll', tablets are packed in transparent pouches for each client by administration time. Each pouch includes a description of which medication it contains. The pouches are delivered sealed together in the form of a medication roll for each client.[80]

3.4. The patient is having trouble storing the medicines properly

3.4.1. Inform the patient about the *conditions* under which the drugs should be stored to maintain their effectiveness (e.g. storage in the refrigerator, in a dark environment, a moisture-free environment). Storage instructions are stated on the packaging and/or package leaflet for each drug. In addition, inform the patient that the drugs should be kept in the original packaging (the packaging supplied by the pharmacy) and with the package leaflet attached.[101]

> *Note:*
> - *When using a medication box in which medicines are set out for one week, the medicines for that week can be removed from the original packaging and stored in the medication box. The remaining medicines should be kept in the original packaging until the medication box is refilled.*
> - *For 'drugs on a roll', the drugs should be stored in the sachets supplied by the pharmacy.*

3.4.2. Inform the patient about drugs with limited shelf life (e.g. eye drops or magistral preparations).[101] Advise patient to write the expiry date on the packaging upon opening it.[80]

3.4.3. Advise patients to check at regular intervals the expiry dates of the drugs they have at home (or have them checked) and to return expired drugs or unused drugs without the packaging and leaflet to the pharmacist.[101]

4. Medication intake

4.1. The patient has swallowing problems

4.1.1. Advise the patient to swallow with the chin on the chest (chin-tuck technique).[80,102-104] An instructional video is available at: https://www.apotheek.nl/instructies/slikken-van-medicijnen/alternatieve-methode.

4.1.2. Determine whether more suitable oral administration forms are available (e.g. liquid form, effervescent tablets, powder).[105,106] Consult the pharmacist, Oralia VTGM of KNMP (NL) or the medication-crushing sheets (BE).[106]

4.1.3. If necessary, adjust the medication administration route in consultation with the prescriber, pharmacist and patient.[105]

4.1.4. Dividing, pulverising or dissolving drugs in water can be useful for facilitating intake. However, always check carefully whether the medicine is suitable for being divided, pulverised or dissolved in water.[106] Consult the pharmacist, Oralia VTGM of KNMP (NL) or the medication-crushing sheets (BE) before issuing this recommendation to the patient.

4.1.5. If swallowing medicines using water is unsuccessful, the use of a swallowing solution (e.g. MediSpend) may be considered or, in consultation with the pharmacist, determine whether the medicine can be taken with food (e.g. yoghurt or apple sauce).[104]

4.1.6. If necessary, refer patients to a speech therapist with known experience in treating swallowing disorders.[105]

4.2. The patient is not using the medicines properly

The patient is taking the medicines at the wrong times

See Section 3.3: The patient is having trouble establishing a daily routine.

The patient is taking incorrect doses (under-dosing or over-dosing, extra doses, forgotten doses)

4.2.1. Advise the patient to consult the drug package leaflet and/or a physician or pharmacist if a dose has been forgotten (**ExpB**).

4.2.2. Advise the patient never to take a double dose of the medicine to make up for a forgotten dose, unless specifically stated otherwise in the package leaflet or if otherwise advised by the physician/pharmacist (**ExpB**).

4.2.3. Advise patients to try to contact the prescriber of the drug in the first instance if they exhibit a reaction to drugs or if too much of a drug has been taken. Patients can also contact the GP, the GP service, the community pharmacist or the emergency department (112). In Belgium, patients can also contact the Belgian Poison Centre for advice in case of drug overdose (070/245.245) (**ExpB**).

The patient is using an improper administration route or technique

4.2.4. Indicate the route of administration on the medication schedule.[78,79,82,83]

4.2.5. Avoid professional jargon (e.g. oral, nasal, cutaneous), and use simple language (e.g. by mouth, in the nose, on the skin).[93,94]

4.2.6. If necessary, use pictograms to visualise the route of administration. Pictograms are available through bijsluiterinbeeld.nl (**ExpB**).

4.2.7. Provide instructions and advice on correct inhalation technique, nasal spray technique, administration of eye drops, injection technique and so forth.[78]

4.2.8. Use the ‘teach-back’ method: Ask the patient to demonstrate the administration method or technique.[74,78,85-87]

4.3. The patient does not want to take the medicines

4.3.1. Ask patients what they know and understand about their drugs. Ask patient about their beliefs concerning medication, about specific concerns or doubts with regard to drugs.[74] Engage with the patient to address concerns and reasons for not wanting to take drugs.[74,81] Valid and reliable instruments are available for asking patients about their beliefs concerning medication (see Appendix B).[107]

4.3.2. If a patient is not taking the drugs because of beliefs about medication:

- Use motivating conversational techniques to change negative opinions and beliefs that patients have about the drugs.[108-110] Recommendations for conducting a motivating interview are presented in Appendix C.
- Discuss the advantages of taking medication.[81]

4.3.3. If a patient is not taking the drugs because of a bad taste, an alternative form or method of administration can be considered in consultation with the pharmacist/prescriber (**ExpB**).

4.3.4. If a patient is not taking the drugs because of side effects:

- In consultation with physician/pharmacist and patient, consider whether the medication regimen can be adjusted to reduce side effects.[81]
- In consultation with physician/pharmacist and patient, review ways to manage side effects.[81]

5. Supervision of desired and adverse effects of medication

5.1. The patient lacks knowledge about the desired and adverse effects (side effects and interactions) of the drugs

5.1.1. Discuss the advantages and long-term effects of the drugs with the patient.[74]

5.1.2. Discuss concerns about the occurrence of adverse effects with the patient and assess the patient's need for information about side effects and interactions of medication. If desired, provide information about the most frequently occurring interactions and side effects.[78,99]

- How side effects and interactions can be avoided
- How to recognise side effects
- What to do when side effects occur (see Section 5.2)
- When it is necessary to contact a healthcare provider (see Section 5.2)

> *Note: Try to coordinate the amount of information to the patient's needs. Do not overload a patient with information on adverse effects if the patient does not need this information. If too much information is provided, there is a risk that the patient will focus on possible adverse effects or not take the drugs. For this reason, limit the information to the most common and relevant adverse effects and alarm symptoms.*

5.1.3. Indicate where patients can find reliable information on side effects, interactions with food and drink, precautionary (or other) measures (e.g. the drug package leaflet, the pharmacist, the GP) (**ExpB**).

5.2. The patient is having trouble taking appropriate measures when side effects occur

5.2.1. Discuss options for dealing with side effects with the patient. Ask patients in what way they would like to deal with side effects if several alternatives were to be available.[74]

5.2.2. Some side effects result from the improper use of drugs. Work with the patient to explore whether the drugs are being taken as prescribed (e.g. with food), and review specific instructions for drug intake with the patient (**ExpB**).

5.2.3. In case of stomach discomfort, advise the patient to take the drug during or just after meals. However, some drugs should be taken on an empty stomach (e.g. thyroid hormone, bisphosphonates). If in doubt, always consult the package leaflet, physician or pharmacist before giving this advice (**ExpB**).

5.2.4. Advise patients to contact the GP or pharmacist in case of hypersensitivity or allergic reactions, such as the occurrence of itching and rash (**ExpB**).

5.2.5. Advise patients to contact the GP or pharmacist in case of severe or persistent symptoms that occur after taking medicine **(ExpB).**

5.2.6. Inform patients that adjusting the drug regimen or discontinuing drug intake should always be done in consultation with the GP or pharmacist (**ExpB**).

6. Maintaining correct and safe medication intake

6.1. The patient has discontinued taking the medication prematurely

6.1.1. Regularly discuss with patients medication adherence, difficulties regarding drug usage and reasons for discontinuing drug intake prematurely.

- In consultation with the patient, consider whether failure to take drugs (or failure to take them correctly) is due to practical problems with drugs or because of perceptual barriers (e.g. certain beliefs and concerns about medication).[74,111] Depending on the difficulties identified, different recommendations from the guidance document can be applied.
- Use motivating conversational techniques.[108,109,111] Recommendations related to conducting a motivating conversation are presented in Appendix C.

6.1.2. Encourage patients to report problems regarding drug usage to the prescriber, GP, pharmacist and/or home nurse (**ExpB**).

PROJECT GROUP

Laura Mortelmans

PhD researcher, Department of Nursing and Midwifery Sciences, Centre for Research and Innovation in Care, Nurse and Pharmaceutical Care, Faculty of Medicine and Health Sciences, University of Antwerp/Research Foundation Flanders, Belgium

Prof. Tinne Dilles

Professor of Nursing, Department of Nursing and Midwifery Sciences, Centre for Research and Innovation in Care, Nurse and Pharmaceutical Care, Faculty of Medicine and Health Sciences, University of Antwerp, Belgium

Prof. Eva Goossens

Professor of Nursing, Department of Nursing and Midwifery Sciences, Centre for Research and Innovation in Care, Nurse and Pharmaceutical Care, Faculty of Medicine and Health Sciences, University of Antwerp/Professor of Nursing, Department of Public Health and Primary Care, KU Leuven/Scientific consultant, Antwerp University Hospital, Belgium

Prof. Mirko Petrovic

Clinical Head of Geriatrics, Ghent University Hospital/Full Professor of Geriatrics and Clinical Pharmacology, Department of Internal Medicine and Paediatrics, Ghent University, Belgium

Prof. Patricia van den Bemt

Hospital Pharmacist-Clinical Pharmacologist-Epidemiologist, Professor of Clinical Pharmacy and Pharmacology, UMCG Groningen, the Netherlands

Prof. Anne-Marie De Cock

Head of the Geriatrics Department, Antwerp Hospital Network/Professor of Geriatrics, Department of Family Medicine, University of Antwerp, Belgium

EXPERT PANEL

Prof. Hein Heidbuchel

Head of the Cardiology Department, Antwerp University Hospital/Full Professor, University of Antwerp, Belgium

A.J. (Arend) Arends

Clinical Geriatrician, Maasstad Hospital Rotterdam, the Netherlands

Dr H.J. (Jeroen) Derijks

Hospital Pharmacist, Clinical Pharmacologist, Head of the Pharmacy Department, Jeroen Bosch Hospital, the Netherlands

Nikki Noorda

Clinical Geriatrician, Clinical Pharmacologist, Dijklander Hospital Hoorn/Purmerend, the Netherlands

Stéphanie Wuyts

Clinical Pharmacist, Pharmacy Department, UZ Brussel, Belgium

David Jansen

Clinical Geriatrician, Clinical Pharmacologist. Radboud UMC Department of Clinical Geriatrics, Nijmegen, the Netherlands

Prof. Veronique Verhoeven

Family Medicine and Population Health (FAMPOP), University of Antwerp, Belgium

Edgard Weening

Lecturer in Pharmacotherapy and Pharmacy Organisation, University of Groningen, the Netherlands

Chiara Aerts

Adjunct Head Nurse, Centre for Cardiovascular Medicine, Imelda Hospital Bonheiden

Prof. Koen Boussery

Pharmaceutical Care Unit, Ghent University, Belgium

Marjan De Graef

Nurse, Clinical Nurse & Paramedical Research and Development, Department of Nursing and Paramedical Sciences, VITAZ, Belgium

Dr N.E. Dijkstra

Nurse, University College Lecturer in Pharmacology, Senior Researcher for the Lectorate Innovation in Healthcare Processes in Pharmacy, Utrecht University of Applied Sciences, Utrecht, the Netherlands/International Board Member of NuPhaC.

Carola Schol (MSc)

Nursing Researcher, Intensive Care Nurse, Erasmus MC Rotterdam, the Netherlands

Dr H.A.W. van Onzenoort

Hospital Pharmacist and Head of the Radboud UMC Pharmacy Department, Nijmegen, the Netherlands

Dr Peter Dieleman

Teaching Assistant, Family Medicine and Population Health Department(FAMPOP), Centre for General Practice, University of Antwerp, Belgium

Nanet De Haas

Hospital Pharmacist, Antwerp University Hospital, Belgium

Dr Femke Ariën

Geriatrician, Antwerp Hospital Network, Belgium

Prof. Nathalie van der Velde

Clinical Geriatrician/Geriatric Internist, Amsterdam UMC, the Netherlands

REFERENCES

1. Barlow J, Wright C, Sheasby J, Turner A, Hainsworth J. Self-management approaches for people with chronic conditions: a review. *Patient education and counseling.* 2002;48(2):177-187.
2. Bailey SC, Oramasionwu CU, Wolf MS. Rethinking adherence: a health literacy-informed model of medication self-management. *J Health Commun.* 2013;18 Suppl 1(Suppl 1):20-30.
3. Cheen MHH, Tan YZ, Oh LF, Wee HL, Thumboo J. Prevalence of and factors associated with primary medication non-adherence in chronic disease: A systematic review and meta-analysis. *Int J Clin Pract.* 2019;73(6):e13350.
4. van Esch Thamar E.M., Brabers Anne E.M., van Dijk Christel, Groenewegen Peter P., de Jong Judith D. *Inzicht in zorgmijden. Aard, omvang, redenen en achtergrondkenmerken.* Nederland: NIVEL;2015.
5. Mortelmans L, De Baetselier E, Goossens E, Dilles T. What Happens after Hospital Discharge? Deficiencies in Medication Management Encountered by Geriatric Patients with Polypharmacy. *International Journal of Environmental Research and Public Health.* 2021;18(13):7031.
6. Davis TC, Wolf MS, Bass PF, 3rd, et al. Literacy and misunderstanding prescription drug labels. *Annals of internal medicine.* 2006;145(12):887-894.
7. Davis TC, Wolf MS, Bass PF, 3rd, et al. Low literacy impairs comprehension of prescription drug warning labels. *Journal of general internal medicine.* 2006;21(8):847-851.
8. Gustafsson J, Kälvemark S, Nilsson G, Nilsson JL. Patient information leaflets--patients' comprehension of information about interactions and contraindications. *Pharmacy world & science : PWS.* 2005;27(1):35-40.
9. Wolf MS, Curtis LM, Waite K, et al. Helping patients simplify and safely use complex prescription regimens. *Archives of internal medicine.* 2011;171(4):300-305.
10. Spiers MV, Kutzik DM, Lamar M. Variation in medication understanding among the elderly. *American journal of health-system pharmacy : AJHP : official journal of the American Society of Health-System Pharmacists.* 2004;61(4):373-380.
11. Gallagher R, Warwick M, Chenoweth L, Stein-Parbury J, Milton-Wildey K. Medication knowledge, adherence and predictors among people with heart failure and chronic obstructive pulmonary disease. *Journal of Nursing and Healthcare of Chronic Illness.* 2011;3(1):30-40.
12. Romero-Sanchez J, Garcia-Cardenas V, Abaurre R, Martínez-Martínez F, Garcia-Delgado P. Prevalence and predictors of inadequate patient medication knowledge. *Journal of evaluation in clinical practice.* 2016;22(5):808-815.
13. Sino CG, Sietzema M, Egberts TC, Schuurmans MJ. Medication management capacity in relation to cognition and self-management skills in older people on polypharmacy. *The journal of nutrition, health & aging.* 2014;18(1):44-49.
14. Dijkstra NE, Sino CGM, Schuurmans MJ, Schoonhoven L, Heerdink ER. Medication self-management: Considerations and decisions by older people living at home. *Research in social & administrative pharmacy : RSAP.* 2020.
15. Caldeira D, Vaz-Carneiro A, Costa J. The impact of dosing frequency on medication adherence in chronic cardiovascular disease: systematic review and meta-analysis. *Revista portuguesa de cardiologia : orgao oficial da Sociedade Portuguesa de Cardiologia = Portuguese journal of cardiology : an official journal of the Portuguese Society of Cardiology.* 2014;33(7-8):431-437.
16. Coleman CI, Limone B, Sobieraj DM, et al. Dosing frequency and medication adherence in chronic disease. *Journal of managed care pharmacy : JMCP.* 2012;18(7):527-539.
17. Carli Lorenzini G. Managing Multiple Medications and Their Packaging for Older People in Home Care Nursing: An Interview Study. *Healthcare.* 2021;9(10):1265.
18. Carli Lorenzini G, Hellström D. Medication Packaging and Older Patients: A Systematic Review. *Packaging Technology and Science.* 2017;30(8):525-558.

19. Notenboom K, Beers E, van Riet-Nales DA, et al. Practical Problems with Medication Use that Older People Experience: A Qualitative Study. *Journal of the American Geriatrics Society.* 2014;62(12):2339-2344.
20. Rowson J, Sangrar A, Rodriguez-Falcon E, et al. Rating Accessibility of Packaging: A Medical Packaging Example. *Packaging Technology and Science.* 2014;27(7):577-589.
21. Ward J, Buckle P, John Clarkson P. Designing packaging to support the safe use of medicines at home. *Applied Ergonomics.* 2010;41(5):682-694.
22. Beckman A, Bernsten C, Parker MG, Thorslund M, Fastbom J. The Difficulty of Opening Medicine Containers in Old Age: A Population-Based Study. *Pharmacy World and Science.* 2005;27(5):393-398.
23. Vlieland ND, van den Bemt BJF, Bekker CL, Bouvy ML, Egberts TCG, Gardarsdottir H. Older Patients' Compliance with Drug Storage Recommendations. *Drugs & aging.* 2018;35(3):233-241.
24. Wieczorkiewicz SM, Kassamali Z, Danziger LH. Behind closed doors: medication storage and disposal in the home. *The Annals of pharmacotherapy.* 2013;47(4):482-489.
25. Mager DR. Medication errors and the home care patient. *Home healthcare nurse.* 2007;25(3):151-155; quiz 156-157.
26. Mira JJ, Lorenzo S, Guilabert M, Navarro I, Pérez-Jover V. A systematic review of patient medication error on self-administering medication at home. *Expert opinion on drug safety.* 2015;14(6):815-838.
27. Yeaw J, Benner JS, Walt JG, Sian S, Smith DB. Comparing adherence and persistence across 6 chronic medication classes. *Journal of managed care pharmacy : JMCP.* 2009;15(9):728-740.
28. McHorney CA, Spain CV. Frequency of and reasons for medication non-fulfillment and non-persistence among American adults with chronic disease in 2008. *Health expectations : an international journal of public participation in health care and health policy.* 2011;14(3):307-320.
29. Vrijens B, De Geest S, Hughes DA, et al. A new taxonomy for describing and defining adherence to medications. *British journal of clinical pharmacology.* 2012;73(5):691-705.
30. Lehane E, McCarthy G. Intentional and unintentional medication non-adherence: a comprehensive framework for clinical research and practice? A discussion paper. *Int J Nurs Stud.* 2007;44(8):1468-1477.
31. Wroe AL. Intentional and Unintentional Nonadherence: A Study of Decision Making. *Journal of Behavioral Medicine.* 2002;25(4):355-372.
32. Cross AJ, Elliott RA, Petrie K, Kuruvilla L, George J. Interventions for improving medication-taking ability and adherence in older adults prescribed multiple medications. *The Cochrane database of systematic reviews.* 2020;5(5):Cd012419.
33. Marcum ZA, Gellad WF. Medication adherence to multidrug regimens. *Clin Geriatr Med.* 2012;28(2):287-300.
34. Mitchell B, Chong C, Lim WK. Medication adherence 1 month after hospital discharge in medical inpatients. *Internal medicine journal.* 2016;46(2):185-192.
35. Coleman EA, Smith JD, Raha D, Min SJ. Posthospital medication discrepancies: prevalence and contributing factors. *Archives of internal medicine.* 2005;165(16):1842-1847.
36. Simpson SH, Eurich DT, Majumdar SR, et al. A meta-analysis of the association between adherence to drug therapy and mortality. *BMJ (Clinical research ed).* 2006;333(7557):15-15.
37. Iuga AO, McGuire MJ. Adherence and health care costs. *Risk Manag Healthc Policy.* 2014;7:35-44.
38. Scales DC, Fischer HD, Li P, et al. Unintentional Continuation of Medications Intended for Acute Illness After Hospital Discharge: A Population-Based Cohort Study. *Journal of general internal medicine.* 2016;31(2):196-202.
39. Cutler RL, Fernandez-Llimos F, Frommer M, Benrimoj C, Garcia-Cardenas V. Economic impact of medication non-adherence by disease groups: a systematic review. *BMJ open.* 2018;8(1):e016982.

40. van Boven JF, Chavannes NH, van der Molen T, Rutten-van Molken MP, Postma MJ, Vegter S. Clinical and economic impact of non-adherence in COPD: a systematic review. *Respiratory medicine.* 2014;108(1):103-113.
41. LeBlanc RG, Choi J. Optimizing medication safety in the home. *Home healthcare now.* 2015;33(6):313-319.
42. Vanwesemael T, Dilles T, Van Rompaey B, Boussery K. An Evidence-Based Procedure for Self-Management of Medication in Hospital: Development and Validation of the SelfMED Procedure. *Pharmacy (Basel, Switzerland).* 2018;6(3).
43. Wright J, Emerson A, Stephens M, Lennan E. Hospital inpatient self-administration of medicine programmes: a critical literature review. *Pharmacy world & science : PWS.* 2006;28(3):140-151.
44. Richardson SJ, Brooks HL, Bramley G, Coleman JJ. Evaluating the effectiveness of self-administration of medication (SAM) schemes in the hospital setting: a systematic review of the literature. *PloS one.* 2014;9(12):e113912.
45. Tran T, Elliott RA, Taylor SE, Woodward MC. A Self-Administration of Medications Program to Identify and Address Potential Barriers to Adherence in Elderly Patients. *The Annals of pharmacotherapy.* 2011;45(2):201-206.
46. Lam P, Elliott RA, George J. Impact of a self-administration of medications programme on elderly inpatients' competence to manage medications: a pilot study. *Journal of clinical pharmacy and therapeutics.* 2011;36(1):80-86.
47. Grantham G, McMillan V, Dunn SV, Gassner LA, Woodcock P. Patient self-medication--a change in hospital practice. *Journal of clinical nursing.* 2006;15(8):962-970.
48. NHS. *Guidelines for Patient Self-Administration of Medication (SAM).* 2014.
49. Masnoon N, Shakib S, Kalisch-Ellett L, Caughey GE. What is polypharmacy? A systematic review of definitions. *BMC geriatrics.* 2017;17(1):230.
50. Midão L, Giardini A, Menditto E, Kardas P, Costa E. Polypharmacy prevalence among older adults based on the survey of health, ageing and retirement in Europe. *Archives of gerontology and geriatrics.* 2018;78:213-220.
51. van den Akker M, Vaes B, Goderis G, Van Pottelbergh G, De Burghgraeve T, Henrard S. Trends in multimorbidity and polypharmacy in the Flemish-Belgian population between 2000 and 2015. *PloS one.* 2019;14(2):e0212046.
52. Hellemans L, Nuyts S, Hias J, et al. Polypharmacy and excessive polypharmacy in community-dwelling middle aged and aged adults between 2011 and 2015. *International Journal of Clinical Practice.* 2021;75(4):e13942.
53. De Staat van Volksgezondheid en Zorg. Polyfarmacie: aantal patiënten. https://www.staatvenz.nl/kerncijfers/polyfarmacie-aantal-pati%C3%ABnten. Published 2021. Updated 16 Mei 2022. Accessed 25/07/2022.
54. Rijksinstituut voor Volksgezondheid en Milieu (RIVM). *Polyfarmacie bij kwetsbare ouderen: inventarisatie van risico's en mogelijke interventiestrategieën* Nederland2013.
55. Cadogan CA, Ryan C, Hughes CM. Appropriate Polypharmacy and Medicine Safety: When Many is not Too Many. *Drug safety.* 2016;39(2):109-116.
56. Aggarwal P, Woolford SJ, Patel HP. Multi-Morbidity and Polypharmacy in Older People: Challenges and Opportunities for Clinical Practice. *Geriatrics (Basel).* 2020;5(4):85.
57. Viktil KK, Blix HS, Eek AK, Davies MN, Moger TA, Reikvam A. How are drug regimen changes during hospitalisation handled after discharge: a cohort study. *BMJ open.* 2012;2(6).
58. Garcia-Caballos M, Ramos-Diaz F, Jimenez-Moleon JJ, Bueno-Cavanillas A. Drug-related problems in older people after hospital discharge and interventions to reduce them. *Age and ageing.* 2010;39(4):430-438.
59. Garin N, Sole N, Lucas B, et al. Drug related problems in clinical practice: a cross-sectional study on their prevalence, risk factors and associated pharmaceutical interventions. *Scientific Reports.* 2021;11(1):883.
60. Cua YM, Kripalani S. Medication use in the transition from hospital to home. *Annals of the Academy of Medicine, Singapore.* 2008;37(2):136-136.

61. Karapinar-Carkit F, Borgsteede SD, Zoer J, Smit HJ, Egberts AC, van den Bemt PM. Effect of medication reconciliation with and without patient counseling on the number of pharmaceutical interventions among patients discharged from the hospital. *The Annals of pharmacotherapy.* 2009;43(6):1001-1010.
62. Daliri S, Bekker CL, Buurman BM, Scholte Op Reimer WJM, van den Bemt BJF, Karapinar-Carkit F. Barriers and facilitators with medication use during the transition from hospital to home: a qualitative study among patients. *BMC Health Serv Res.* 2019;19(1):204.
63. Bagge M, Norris P, Heydon S, Tordoff J. Older people's experiences of medicine changes on leaving hospital. *Research in social & administrative pharmacy : RSAP.* 2014;10(5):791-800.
64. Harris CM, Sridharan A, Landis R, Howell E, Wright S. What happens to the medication regimens of older adults during and after an acute hospitalization? *Journal of patient safety.* 2013;9(3):150-153.
65. Knight DA, Thompson D, Mathie E, Dickinson A. 'Seamless care? Just a list would have helped!' Older people and their carer's experiences of support with medication on discharge home from hospital. *Health expectations : an international journal of public participation in health care and health policy.* 2013;16(3):277-291.
66. Ziaeian B, Araujo KL, Van Ness PH, Horwitz LI. Medication reconciliation accuracy and patient understanding of intended medication changes on hospital discharge. *Journal of general internal medicine.* 2012;27(11):1513-1520.
67. Pasina L, Brucato AL, Falcone C, et al. Medication non-adherence among elderly patients newly discharged and receiving polypharmacy. *Drugs & aging.* 2014;31(4):283-289.
68. Schoonover H, Corbett CF, Weeks DL, Willson MN, Setter SM. Predicting potential postdischarge adverse drug events and 30-day unplanned hospital readmissions from medication regimen complexity. *Journal of patient safety.* 2014;10(4):186-191.
69. Elliott RA, Marriott JL. Standardised assessment of patients' capacity to manage medications: a systematic review of published instruments. *BMC geriatrics.* 2009;9:27.
70. Farris KB, Phillips BB. Instruments assessing capacity to manage medications. *The Annals of pharmacotherapy.* 2008;42(7):1026-1036.
71. Advinha AM, Lopes MJ, de Oliveira-Martins S. Assessment of the elderly's functional ability to manage their medication: a systematic literature review. *International journal of clinical pharmacy.* 2017;39(1):1-15.
72. Badawoud AM, Salgado TM, Lu J, Parsons P, Peron EP, Slattum PW. Measuring Medication Self-Management Capacity: A Scoping Review of Available Instruments. *Drugs & aging.* 2020;37(7):483-501.
73. Hsu C-C, Sandford BA. The Delphi technique: making sense of consensus. *Practical assessment, research, and evaluation.* 2007;12(1):10.
74. Nunes V NJ, O'Flynn N, Calvert N, Kuntze S, Smithson H, et al. *Clinical Guidelines and Evidence Review for Medicines Adherence: involving patients in decisions about prescribed medicines and supporting adherence.* London: National Collaborating Centre for Primary Care and Royal College of General Practitioners.;2009.
75. Rijksinstituut voor ziekte- en invaliditeitsverzekering (RIZIV). 'Goedkoopste' geneesmiddelen. RIZIV. https://www.riziv.fgov.be/nl/toepassingen/Paginas/goedkoopste-geneesmiddelen.aspx. Updated 31/01/2022. Accessed 15/02/2022.
76. Rijksinstituut voor ziekte- en invaliditeitsverzekering (RIZIV). 'Goedkoop voorschrijven'. https://www.riziv.fgov.be/nl/professionals/individuelezorgverleners/artsen/verzorging/Paginas/goedkoop-voorschrijven-20150101.aspx. Updated 13/10/2021. Accessed 14/03/2022.
77. Rijksinstituut voor ziekte- en invaliditeitsverzekering (RIZIV). Voorschijven op stofnaam: regels voor de apotheker voor het uitvoeren van het voorschrift. https://www.riziv.fgov.be/nl/themas/kost-terugbetaling/door-ziekenfonds/geneesmiddel-gezondheidsproduct/afleveren/Paginas/voorschijven-stofnaam-regels-apotheker-uitvoeren-voorschrift.aspx. Updated 30/06/2020. Accessed 14/07/2022, 2022.
78. Belgische Vereniging voor Gerontologie en Geriatrie. *RICHTLIJN: HOE MEDICATIEBEGELEIDING ("MEDICATIE COUNSELLING") BIJ OUDERE VOLWASSEN UIT TE VOEREN BIJ ZIEKENHUISONTSLAG.* 2020.

79. Scottish Intercollegiate Guidelines Network (SIGN). The SIGN discharge document. In. Edinburgh: SIGN; 2012.
80. Instituut Verantwoord Medicijngebruik. Tips voor hulpmiddelen en hulp beheer eigen medicatie. https://www.medicijngebruik.nl/zorginstellingen/werkmateriaal-overig/2118. Updated 06/02/2020. Accessed 15/02/2022.
81. Marek KD, Antle L. Advances in Patient Safety. Medication Management of the Community-Dwelling Older Adult. In: Hughes RG, ed. *Patient Safety and Quality: An Evidence-Based Handbook for Nurses.* Rockville (MD): Agency for Healthcare Research and Quality (US); 2008.
82. National Institute for Health and Care Excellence. Medicines optimisation: the safe and effective use of medicines to enable the best possible outcomes. NICE. https://www.nice.org.uk/guidance/ng5. Published 2015. Accessed 13/07/2021.
83. Dietrich FM, Hersberger KE, Arnet I. Benefits of medication charts provided at transitions of care: a narrative systematic review. *BMJ open.* 2020;10(10):e037668.
84. Koninklijke Nederlandse Maatschappij ter bevordering der Pharmacie. Overdracht van Medicatiegegevens in de keten. https://www.knmp.nl/index.php/richtlijnen/overdracht-van-medicatiegegevens-de-keten. Updated 10/01/2022. Accessed 16/07/2022.
85. Brega AG, Barnard J, Mabachi NM, et al. *AHRQ Health Literacy Universal Precautions Toolkit, Second Edition. .* Agency for Healthcare Research and Quality;2015.
86. Nederlandse Vereniging voor Klinische Geriatrie (NVKG). Polyfarmacie bij ouderen. https://richtlijnendatabase.nl/richtlijn/polyfarmacie_bij_ouderen/polyfarmacie_bij_ouderen_-_korte_beschrijving.html. Updated 03/12/2020. Accessed 07/03/2022.
87. Ha Dinh TT, Bonner A, Clark R, Ramsbotham J, Hines S. The effectiveness of the teach-back method on adherence and self-management in health education for people with chronic disease: a systematic review. *JBI database of systematic reviews and implementation reports.* 2016;14(1):210-247.
88. Rijksinstituut voor ziekte- en invaliditeitsverzekering (RIZIV). Begeleiden van chronische patiënten als huisapotheker. https://www.riziv.fgov.be/nl/professionals/individuelezorgverleners/apothekers/Paginas/huisapotheker-begeleiden-chronische-patienten.aspx. Updated 24/01/2022. Accessed.
89. Sharko M, Sharma MM, Benda NC, et al. Strategies to optimize comprehension of numerical medication instructions: A systematic review and concept map. *Patient education and counseling.* 2022.
90. Sletvold H, Sagmo LAB, Torheim EA. Impact of pictograms on medication adherence: A systematic literature review. *Patient education and counseling.* 2020;103(6):1095-1103.
91. Mullen RJ, Duhig J, Russell A, Scarazzini L, Lievano F, Wolf MS. Best-practices for the design and development of prescription medication information: A systematic review. *Patient education and counseling.* 2018;101(8):1351-1367.
92. Wali H, Hudani Z, Wali S, Mercer K, Grindrod K. A systematic review of interventions to improve medication information for low health literate populations. *Research in social & administrative pharmacy : RSAP.* 2016;12(6):830-864.
93. Maghroudi E, van Hooijdonk CMJ, van de Bruinhorst H, van Dijk L, Rademakers J, Borgsteede SD. The impact of textual elements on the comprehensibility of drug label instructions (DLIs): A systematic review. *PLoS One.* 2021;16(5):e0250238.
94. Bailey SC, Navaratnam P, Black H, Russell AL, Wolf MS. Advancing Best Practices for Prescription Drug Labeling. *Ann Pharmacother.* 2015;49(11):1222-1236.
95. Zorg voor beter. Hulpmiddelen voor veilig medicijngebruik. https://www.zorgvoorbeter.nl/medicatieveiligheid/informatie-ouderen/hulpmiddelen. Updated 01/02/2022. Accessed 15/02/2022.
96. Vilans in opdracht van Ministerie van Volksgezondheid Welzijn en Sport. Hulpmiddelenwijzer. https://hulpmiddelenwijzer.nl/hulpmiddelen/bij/zorgen-en-verzorgen/medicijnen-nemen. Accessed 13/04/2022.
97. Simas da Rocha B, Garcia Moraes C, Miyake Okumura L, da Cruz F, Sirtori L, da Silva Pons E. Interventions to Reduce Problems Related to the Readability and Comprehensibility of Drug Packages and Labels: A Systematic Review. *Journal of patient safety.* 2021;17(8).

98. Shrank W, Avorn J, Rolon C, Shekelle P. Effect of content and format of prescription drug labels on readability, understanding, and medication use: a systematic review. *The Annals of pharmacotherapy.* 2007;41(5):783-801.
99. Ryan R, Santesso N, Lowe D, et al. Interventions to improve safe and effective medicines use by consumers: an overview of systematic reviews. *The Cochrane database of systematic reviews.* 2014;2014(4):Cd007768.
100. Conn VS, Ruppar TM, Enriquez M, Cooper P. Medication adherence interventions that target subjects with adherence problems: Systematic review and meta-analysis. *Research in social & administrative pharmacy : RSAP.* 2016;12(2):218-246.
101. Federaal agentschap voor geneesmiddelen en gezondheidsproducten (FAGG). Geneesmiddelen bewaren. https://www.fagg-afmps.be/nl/info_patienten/geneesmiddelen_bewaren. Updated 16/12/2020. Accessed 15/02/2022.
102. De Bodt M, Guns C, D'Hondt M, Vanderwegen J, Van Nuffelen G. *Dysfagie. Handboek voor de klinische praktijk.* Garant; 2015.
103. Farmaka. Slikstoornissen bij ouderen: fysiologie, pathologie en aanpak. J. Lannoy. https://farmaka.bcfi.be/nl/formulariuminfo/brief/slikstoornissen-bij-ouderen-fysiologie-pathologie-en-aanpak. Published 2017. Accessed 14/03/2022.
104. Koninklijke Nederlandse Maatschappij ter bevordering der Pharmacie. Moeite met slikken van medicijnen. https://www.apotheek.nl/zorg-van-de-apotheker/juist-medicijngebruik-is-van-levensbelang/moeite-met-slikken-van-medicijnen. Updated 07-11-2022. Accessed 14/03/2022.
105. Nederlandse Vereniging voor Keel–Neus–Oorheelkunde en Heelkunde van het Hoofd–Halsgebied (NVKVO). Orofaryngeale dysfagie Federatie Medisch Specialisten. https://richtlijnendatabase.nl/richtlijn/orofaryngeale_dysfagie/medicatie_bij_dysfagie.html. Updated 01/02/2017. Accessed 14/03/2022.
106. Farmaka. Delen of pletten van geneesmiddelen. https://farmaka.bcfi.be/nl/formularium/390#main. Accessed 04/03/2022.
107. Horne R, Weinman J, Hankins M. The Beliefs about Medicines Questionnaire: The development and evaluation of a new method for assessing the cognitive representation of medication. *Psychology & Health.* 1999;14(1):1-24.
108. Palacio A, Garay D, Langer B, Taylor J, Wood BA, Tamariz L. Motivational Interviewing Improves Medication Adherence: a Systematic Review and Meta-analysis. *Journal of general internal medicine.* 2016;31(8):929-940.
109. Zomahoun HTV, Guénette L, Grégoire J-P, et al. Effectiveness of motivational interviewing interventions on medication adherence in adults with chronic diseases: a systematic review and meta-analysis. *International Journal of Epidemiology.* 2017;46(2):589-602.
110. Miller WR, Rollnick S. *Motivational interviewing : preparing people for change.* New York: Guilford Press; 2002.
111. Nederlands Huisartsen Genootschap (NHG), de Koninklijke Nederlandse Maatschappij ter bevordering der Pharmacie (KNMP), Verpleegkundigen en Verzorgenden Nederland (V&VN). *LESA Organisatie van zorg bij chronische medicatie. Handreikingen voor huisartsen, apothekers en wijkverpleging voor het maken van praktische afspraken over de organisatie van zorg rondom medicatiegebruik voor patiënten met wijkverpleging.* Nederland2020.

APPENDICES

Appendix A: List of problems relating to medication self-management

Stage in the medication self-management process (Bailey)	Potential problems based on literature	Cluster
Picking up the medicines at the pharmacy	– The patient cannot afford the drugs.	Financial problems
	– The patient cannot independently pick up the drugs at the pharmacy (e.g. due to lack of transport, poor state of health).<br>– The patient has no prescription (or repeat prescription) available/does not know when to obtain a new prescription.<br>– The patient does not know how to obtain new or repeat prescriptions/drugs (e.g. patient does not know whom to contact).	No supply of medication
Knowledge and understanding of medication	– The patient has no knowledge concerning some or all of the patient's own drugs (e.g. knowledge of name, dosage, timing, administration route and/or indication).	Lack of knowledge concerning drugs
	– The patient is having trouble understanding information and/or instructions concerning the drugs.	Trouble understanding information and/or instructions concerning drugs
Practical organisation and scheduling of medication intake	– The patient is having trouble organising/filling a medication box.<br>– The patient is having trouble opening packaging (e.g. removing tablets/capsules from blister packaging).<br>– The patient is having trouble halving/breaking drugs.	Trouble preparing drugs (or drug intake)
	– The patient is having trouble reading labels/stickers/ information/instructions (e.g. on drug packaging, package leaflet, medication schedule).	Trouble reading labels/stickers/information /instructions

	– The patient is having trouble establishing a daily routine.	Trouble establishing daily routine
	– The patient is having trouble storing drugs properly.	Trouble storing drugs properly
Medication intake	– The patient has swallowing problems.	Swallowing problems
	– The patient does not want to take the medication.	Not wanting to take drugs
	– The patient is taking incorrect doses of drugs (under-dosing, over-dosing, extra doses, forgotten doses).<br>– The patient is not administering the drugs properly.<br>– The patient is taking the drugs at the wrong time of day.	Improper use of drugs
Supervision of desired and adverse effects of medication	– The patient lacks knowledge on side effects, contraindications, interactions and/or precautions.	Lack of knowledge on side effects, interactions and/or precautionary measures
	– The patient lacks knowledge on measures to be taken/does not take appropriate measures in case of side effects or after missing a dose.	Difficulty taking appropriate measures in case of side effects or missed doses
Maintaining correct and safe medication intake	– The patient has discontinued taking the drugs prematurely.	Premature discontinuation of drug intake

Appendix B: Beliefs about medication questionnaire

YOUR VIEWS ABOUT MEDICINES PRESCRIBED FOR YOU

We would like to ask you about your personal views about medicines prescribed for you. These are statements other people have made about their medicines. Please indicate the extent to which you agree or disagree with them by ticking the appropriate box. There are no right or wrong answers. We are interested in your personal views.

	**Strongly disagree**	**Disagree**	**Uncertain**	**Agree**	**Strongly agree**
My health, at present, depends on my medicines.	❑	❑	❑	❑	❑
Having to take medicines worries me	❑	❑	❑	❑	❑
My life would be impossible without my medicines	❑	❑	❑	❑	❑
I sometimes worry about long-term effects of my medicines	❑	❑	❑	❑	❑
Without my medicines I would be very ill	❑	❑	❑	❑	❑
My medicines are a mystery to me	❑	❑	❑	❑	❑
My health in the future will depends on my medicines	❑	❑	❑	❑	❑
My medicines disrupt my life	❑	❑	❑	❑	❑
I sometimes worry about becoming too dependent on my medicines	❑	❑	❑	❑	❑
My medicines protect me from becoming worse	❑	❑	❑	❑	❑

YOUR VIEWS ABOUT MEDICINES IN GENERAL

We would like to ask you about your personal views about medicines in general. These are statements other people have made about medicines in general. Please indicate the extent to which you agree or disagree with them by ticking the There are no right or wrong answers. We are interested in your personal views.

	**Strongly disagree**	**Disagree**	**Uncertain**	**Agree**	**Strongly agree**
Doctors use too many medicines	❑	❑	❑	❑	❑
People who take medicines should stop their treatment for a while every now and again	❑	❑	❑	❑	❑
Most medicines are addictive	❑	❑	❑	❑	❑
Natural remedies are safer than medicines	❑	❑	❑	❑	❑
Medicines do more harm than good	❑	❑	❑	❑	❑
All medicines are poisons	❑	❑	❑	❑	❑
Doctors place too much trust on medicines	❑	❑	❑	❑	❑
If doctors had more time with patients they would prescribe fewer medicines.	❑	❑	❑	❑	❑

© Horne R, Weinman J, Hankins M. The Beliefs about Medicines Questionnaire: The development and evaluation of a new method for assessing the cognitive representation of medication. *Psychology & Health*. 1999;14(1):1-24.

Appendix C: Motivating conversational techniques

Basic information

Motivational interview

- Refers to having an open conversation and listening, with the goal of allowing the patient to self-discover the need for change and to find motivation for making changes
- Especially helpful for starting and sustaining lifestyle changes (e.g. exercising more, losing weight, quitting smoking or stopping alcohol consumption)
- Helps in the application of self-care
- Makes the patient accountable

Practical implementation

- Ask open-ended questions (What? How?).
    - ‘What do you think about your medication?’ or ‘What do you experience with regard to taking your medication?’
    - Avoid questions that the patient can answer with one word (yes/no).
- Show empathy.
    - If patients feel that they are being listened to and that there is consent, it will be easier for them to work on their health.
    - ‘The many side effects that you are experiencing must be difficult. How do you cope with this?’
- Show interest when listening.
    - Glances, gestures and facial expressions, like slight nods or brief affirmations (e.g. ‘yes’).
    - Eye contact is important; do not look at a computer screen.
- Make reflective comments, in which you repeat the patient’s own words.
    - This will encourage the patient to think further or tell you more.
    - ‘Did I understand correctly that you stopped taking your medication before?’
- Recognise and engage with talk about change.
    - Repeat the patient’s own words and ask follow-up questions about them.
    - Help patients to understand the conflict between their current situations and where they would ultimately like to be. It should be the patient who indicates the motives for change, and not the healthcare provider.
    - ‘You say that you are worried about the side effects of your medication. What do you mean by that?’
- Adopt a neutral position and do not ‘engage with resistance’.
    - Go along with the patient’s story and avoid arguing and giving direct instructions.
    - Medical argumentation at the beginning of the discussion can lead to resistance.
    - Make patients think for themselves, find new angles and make the situation clear to them.
- Emphasise what is positive and successful.
    - Emphasise the positive things that the patient shares. Try to grasp onto the smallest positive thing. Compliment any success.
- Reinforce the ability to change and emphasise the patient’s power and potential to change (empowerment).
    - Find the patient’s strengths and enhance their belief in their own possibilities
    - ‘You seem very determined.’

    - Patients will choose for themselves how they would like to change.
- Considering the advantages and disadvantages can be very helpful if a patient is uncertain about the change.
    - Have patients sum up the advantages and disadvantages themselves.
    - ‘Which positive aspects does taking the drugs properly have?’
    - ‘What advantage could you realise by taking drugs the properly?’
    - At this point, you can use neutral affirmation (e.g. ‘You’re right, taking medicine properly ...’).
- Summarise briefly and repeat.
    - Use the patient’s own words and phrasing.
    - This reinforces the sense of being heard and the possibility of change.
- The healthcare provider is a medical expert.
    - You do not need to withhold information; just wait for an appropriate moment during the interview.
    - A conversation will make the patient reflect.

Source:
Mustajoki P, Alenius H. De rol van een motivationeel interview bij het veranderen van levensstijl en behandeling [The role of a motivational interview in changing lifestyle and treatment]. ebpracticenet. Available from https://ebpnet.be/nl/ebsources/1231?searchTerm=de%20rol%20van%20motiv. Updated 27/046/2017. Accessed 13/04/2022
